# Brain energy metabolism: A roadmap for future research

**DOI:** 10.1111/jnc.16032

**Published:** 2024-01-06

**Authors:** Caroline D. Rae, Joseph A. Baur, Karin Borges, Gerald Dienel, Carlos Manlio Díaz-García, Starlette R. Douglass, Kelly Drew, João M. N. Duarte, Jordi Duran, Oliver Kann, Tibor Kristian, Dasfne Lee-Liu, Britta E. Lindquist, Ewan C. McNay, Michael B. Robinson, Douglas L. Rothman, Benjamin D. Rowlands, Timothy A. Ryan, Joseph Scafidi, Susanna Scafidi, C. William Shuttleworth, Raymond A. Swanson, Gökhan Uruk, Nina Vardjan, Robert Zorec, Mary C. McKenna

**Affiliations:** 1 School of Psychology, The University of New South Wales, NSW 2052 & Neuroscience Research Australia, Randwick, New South Wales, Australia; 2 Department of Physiology and Institute for Diabetes, Obesity and Metabolism, Perelman School of Medicine, University of Pennsylvania, Philadelphia, Pennsylvania, USA; 3 School of Biomedical Sciences, Faculty of Medicine, The University of Queensland, St Lucia, QLD, Australia; 4 Department of Neurology, University of Arkansas for Medical Sciences, Little Rock, Arkansas, USA; 5 Department of Cell Biology and Physiology, University of New Mexico School of Medicine, Albuquerque, New Mexico, USA; 6 Department of Biochemistry and Molecular Biology, Center for Geroscience and Healthy Brain Aging, University of Oklahoma Health Sciences Center, Oklahoma City, Oklahoma, USA; 7 Behavioral Neuroscience, University at Albany, Albany, New York, USA; 8 Center for Transformative Research in Metabolism, Institute of Arctic Biology, University of Alaska Fairbanks, Fairbanks, Alaska, USA; 9 Department of Experimental Medical Science, Faculty of Medicine, Lund University, Lund, & Wallenberg Centre for Molecular Medicine, Lund University, Lund, Sweden; 10 Institut Químic de Sarrià (IQS), Universitat Ramon Llull (URL), Barcelona, Spain; 11 Institute for Bioengineering of Catalonia (IBEC), The Barcelona Institute of Science and Technology, Barcelona, Spain; 12 Institute of Physiology and Pathophysiology, University of Heidelberg, D-69120; Interdisciplinary Center for Neurosciences (IZN), University of Heidelberg, Heidelberg, Germany; 13 Veterans Affairs Maryland Health Center System, Baltimore, Maryland, USA; 14 Department of Anesthesiology and the Center for Shock, Trauma, and Anesthesiology Research (S.T.A.R.), University of Maryland School of Medicine, Baltimore, Maryland, USA; 15 Facultad de Medicina y Ciencia, Universidad San Sebastián, Santiago, Región Metropolitana, Chile; 16 Department of Neurology, Division of Neurocritical Care, Gladstone Institute of Neurological Disease, University of California at San Francisco, San Francisco, California, USA; 17 Departments of Pediatrics and System Pharmacology & Translational Therapeutics, Children’s Hospital of Philadelphia, University of Pennsylvania, Philadelphia, Pennsylvania, USA; 18 Magnetic Resonance Research Center and Departments of Radiology and Biomedical Engineering, Yale University, New Haven, Connecticut, USA; 19 School of Chemistry, Faculty of Science, The University of Sydney, Sydney, New South Wales, Australia; 20 Department of Biochemistry, Weill Cornell Medicine, New York, New York, USA; 21 Department of Neurology, Kennedy Krieger Institute, Johns Hopkins University School of Medicine, Baltimore, Maryland, USA; 22 Anesthesiology & Critical Care Medicine, Johns Hopkins University School of Medicine, Baltimore, Maryland, USA; 23 Department of Neurosciences, University of New Mexico School of Medicine Albuquerque, Albuquerque, New Mexico, USA; 24 Department of Neurology, University of California, San Francisco, and San Francisco Veterans Affairs Medical Center, San Francisco, California, USA; 25 Laboratory of Cell Engineering, Celica Biomedical, Ljubljana, Slovenia; 26 Laboratory of Neuroendocrinology—Molecular Cell Physiology, Institute of Pathophysiology, Faculty of Medicine, University of Ljubljana, Ljubljana, Slovenia; 27 Department of Pediatrics and Program in Neuroscience, University of Maryland School of Medicine, Baltimore, Maryland, USA

**Keywords:** acetate, aerobic glycolysis, GLUT4, insulin, noradrenaline

## Abstract

Although we have learned much about how the brain fuels its functions over the last decades, there remains much still to discover in an organ that is so complex. This article lays out major gaps in our knowledge of interrelationships between brain metabolism and brain function, including biochemical, cellular, and subcellular aspects of functional metabolism and its imaging in adult brain, as well as during development, aging, and disease. The focus is on unknowns in metabolism of major brain substrates and associated transporters, the roles of insulin and of lipid droplets, the emerging role of metabolism in microglia, mysteries about the major brain cofactor and signaling molecule NAD^+^, as well as unsolved problems underlying brain metabolism in pathologies such as traumatic brain injury, epilepsy, and metabolic downregulation during hibernation. It describes our current level of understanding of these facets of brain energy metabolism as well as a roadmap for future research.

## 1 | INTRODUCTION

Brain energy metabolism is unique among major organs due to the complexity of the coupling between neuronal and glial metabolism to support the energetic and biosynthesis requirements for neuronal signaling (neurometabolic coupling) and the disproportionate increase in CBF to support increases in neuronal energy requirements (neurovascular coupling). Both neurometabolic and neurovascular coupling are altered in a range of neurological and psychiatric diseases, and even in metabolic syndrome and normal aging, and may have a major role in the associated loss of brain function. Brain energy metabolism is complex because its functional roles can change with maturation, aging, and disease. Metabolic specialization includes fuelling energy-requiring processes, providing substrates for synthesis of macromolecules, maintenance of signaling processes, and support of higher brain functions including cognition and memory consolidation. Specialization involves differences in metabolite concentrations, pathway fluxes, and regulatory processes at the cellular and subcellular level. Enormous progress has been made in all of these areas, but important knowledge gaps remain. Put simply, we still cannot quantitively predict the impact of a pharmaceutical or dietary intervention on normalizing metabolism and associated function, even for diseases with known initial etiologies such as transporter or metabolic enzyme mutations. Bridging this gap was the primary goal of the 14th International Conference on Brain Energy Metabolism titled “Energy substrates and Microbiome govern brain bioenergetics and function with ageing” held in October 24th to October 27th, 2022 at Santa Fe, New Mexico.

The meeting brought together participants with a wide range of expertise who have provided brief overviews of selected topics and unresolved questions for future research which are key for translating advances in basic and translational metabolic research to improve diagnosis and treatment, as well as dietary and other strategies to preserve brain health in aging and metabolic syndrome. To facilitate interactions among researchers interested in pursuing work in the areas identified in this article, the authors of each subsection are identified in the author contributions section so that they can be contacted for further discussions. Major themes are presented in the following sequence:

- Synthesis of glucose-derived neurotransmitters and fuelling neurotransmission
  - How does excitatory/inhibitory signaling ensure its energy supply? Unresolved issues in understanding coupling of neurotransmission with glucose oxidation
  - When and where is glutamate being used to fuel brain energy metabolism?
  - When does brain use the anaplerotic enzyme pyruvate carboxylase and under what conditions?
- Branch pathways to and from the Krebs cycle: Trafficking and compartmentation
  - What are the functions of lactate?
  - Why is the sodium-dependent citrate transporter SLC13A5 important in the brain?
  - Brain acetate metabolism is compartmentalized, but why?
- Energetics of signal processing and higher brain functions
  - The demand–supply problem in neuronal bioenergetics
  - How does brain insulin modulate metabolism, cognition, and memory?
- Redox molecules: Transcriptional regulation, aging, and neurodegeneration
  - What we do not know about NAD^+^ metabolism in the brain
  - Intracellular compartmentation and transport of NAD^+^
- Fatty acid turnover during development, aging, and disease
  - Fatty acid synthesis and oxidation after developmental injury: can we find a balance?
  - Emerging roles of lipid droplets in astrocytes: Neuroprotection against disease progression?
- Metabolic dysfunction in aging
  - Metabolic tuning throughout the anatomy of aging neurons
  - Mitigating cognitive decline through astroglial metabolism: Toward the noradrenergic hypothesis of neurodegeneration
- Brain disorders, injury, and inflammation – metabolism-based therapeutics
  - FDG-PET interpretations and cell type-specific utilization of glucose and auxiliary fuels in health and disease
  - What are the mechanisms that link brain glycogen metabolism and pathology?
  - Static vs. Dynamic Impairment in Energy Metabolism Following Acute Brain Injury
  - Why should we use metabolic network modeling to study metabolic regulation of epigenetics in epilepsy?
  - How do activated microglia affect neuroenergetics and brain function?
  - Why do activated microglia require aerobic glycolysis?
- Metabolic control under extreme conditions
  - How does the CNS modulate the circannual rhythm of metabolism in mammals?

## 2 | SYNTHESIS OF GLUCOSE-DERIVED NEUROTRANSMITTERS AND FUELLING NEUROTRANSMISSION

### 2.1 | How does excitatory/inhibitory signaling ensure its energy supply? Unresolved issues in understanding coupling of neurotransmission with glucose oxidation

**FIGURE 1:**
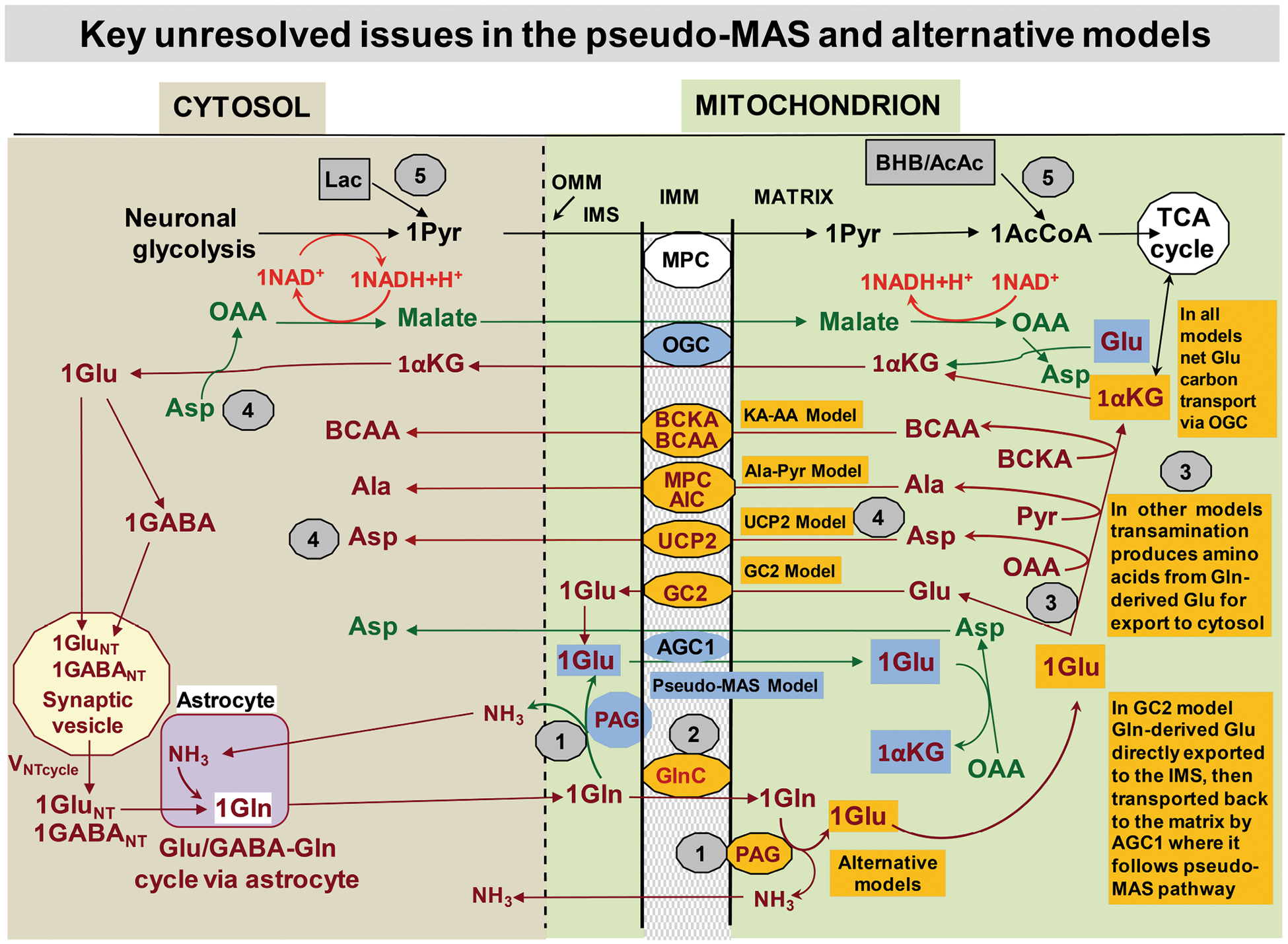
The pseudo-malate–aspartate shuttle (pseudo-MAS) and proposed alternative models for coupling of neuronal glucose oxidation with the Glu/GABA-Gln cycle. The stepwise process of the glutamate/GABA-glutamine (Glu/GABA-Gln) cycle begins with Glu or GABA release from neurons followed by uptake into astrocytes, conversion to Gln that is released, taken up by neurons, converted to Glu/GABA, and incorporated into synaptic vesicles. The pseudo-MAS model illustrated by the blue-background fill shows Gln converted to Glu by phosphate-activated glutaminase (PAG). PAG was assumed to be located on the cytosolic side of the inner mitochondrial membrane (IMM; intermembrane space = IMS) to which Glu is accessible because the outer mitochondrial membrane (OMM) is permeable to metabolites (dashed line). The proposed coupling of the Glu-Gln cycle with glucose oxidation required Glu entry into the matrix in exchange for aspartate (Asp) via the aspartate–glutamate carrier 1 (AGC1), a component of the MAS. After Glu transamination, the α-ketoglutarate (αKG) is exchanged with malate and exits to the cytosol via the oxoglutarate carrier (OGC), another major component of the MAS. The cytosolic αKG is converted to Glu by transamination followed by packaging in synaptic vesicles, or Glu is decarboxylated to produce GABA followed by insertion into a vesicle. The overall mechanistic stoichiometry is 1:1:1 for Glu or GABA:Gln:neurotransmitter. Reduction of cytosolic oxaloacetate to malate regenerates NAD^+^, sustains glycolytic flux, and produces pyruvate (Pyr). Pyr enters the matrix via the mitochondrial pyruvate carrier (MPC) for oxidation in the tricarboxylic acid (TCA) cycle via acetyl CoA (AcCoA). To sum up, the pseudo-MAS model proposes transfer of reducing equivalents from the cytosol to mitochondria with generation of the oxidative substrate from glucose, linking glucose oxidation to the Glu/GABA-Gln cycle. Alternative models (orange-background fill) include a Gln carrier (GlnC) to transport Gln to the matrix where it is the substrate for PAG. The Glu can directly exit to the cytosol via the Glu carrier 2 (GC2) or be transaminated to αKG that exits via OGC. Coupling of the GC2 model to AGC1 transport of Glu back into the mitochondria becomes equivalent to the pseudo-MAS model. Various aminotransferases and carriers in alternative models generate and shuttle amino acids that are exported to the cytosol: uncoupling protein 2 (UCP2), MCP and alanine (Ala) carrier (AlC), or branched-chain keto acid (BCKA) and branched-chain amino acid (BCAA) carriers. The grey-background circles with numbers identify key unresolved steps in the Glu/GABA-Gln cycle and its coupling with glucose oxidation (see text). Modified from Figure 1 in (Rothman, Behar, & Dienel, 2022) © 2022 International Society for Neurochemistry, with re-use permission as authors of original figure.

Excitatory glutamatergic neurotransmission is a major mechanism for neuronal signaling that involves an approximate 1:1 mechanistic stoichiometry between the rate of the glutamate/γ-aminobutyric acid-glutamine (Glu/GABA-Gln) cycle and neuronal glucose oxidation (Rothman, Dienel, et al., 2022; Sibson et al., 1998; Yu et al., 2018). Knowledge of the exact details of this process and elucidation of the pathway coupling mechanism(s) are required to improve interpretation of metabolic brain imaging studies of higher brain functions and disease. The Glu/GABA-Gln cycle involves neurotransmitter Glu and GABA trafficking between neurons and astrocytes via Gln (see Figure 1 and legend for details). Palaiologos et al. (Palaiologos et al., 1988) proposed a stoichiometric mechanism, called the pseudo-malate–aspartate shuttle (pseudo-MAS), that links the Glu-Gln cycle with presynaptic glycolysis and oxidation of glucose (Figure 1, blue-filled text boxes). Detailed re-evaluation of the pseudo-MAS mechanism by Rothman, Behar, and Dienel (2022) identified limitations of the pseudo-MAS model and proposed alternative models (Figure 1, orange-filled text boxes) that address unresolved issues requiring further studies but maintain the same mechanistic stoichiometry. In brief, important knowledge gaps related to mechanisms of coupling neurotransmission with oxidative and glycolytic ATP production include, but are not limited to, the following five key issues (Figure 1, numbers in gray-filled circles):

1. *What is the mitochondrial subcellular localization of the functionally active site(s) of phosphate-activated glutaminase (PAG)*? Evidence strongly supports matrix localization, but activity may occur without Glu entering the matrix. Localization is essential for establishing the requirement for a Gln carrier and for Glu and NH_3_ trafficking across the mitochondrial membrane.
2. *How does glutamine enter the mitochondria*? Gln has access to the mitochondrial matrix, but the identity and mechanism of the putative mitochondrial Gln carrier are unknown. This carrier is a major, unidentified component of the Glu-Gln cycle.
3. *What are the transamination reactions needed to transfer amino groups to alpha-ketoglutarate for glutamate synthesis*? Specific transamination reactions involved in keto- and amino acid interconversions are required for the pseudo-MAS, alternative models, and Glu/GABA-Gln cycle, but the major participants in mitochondria and cytosol remain to be identified.
4. *What are major mitochondrial carriers involved in the above shuttling processes*? UCP2 was suggested by Rothman et al. to be the predominant carrier because it transfers Asp from the matrix to cytosol where it can be converted to oxaloacetate and then malate to regenerate NAD^+^ in the cytosol. However, this and other possibilities remain to be directly tested and established.
5. *Is an obligatory need for glycolytic NADH for the pseudo-malate aspartate shuttle the reason why alternate fuels lactate and β-hydroxybutyrate are unable to displace the requirement for glucose consumption to support functional energy metabolism and Glu/GABA neurotransmission*? From the standpoint of the pseudo-MAS, lactate will be able to provide the necessary NADH so that the glucose dependence likely is due to a separate non-displaceable role for glucose metabolism. During activation, the additional need for glucose can be explained by the large increase in non-oxidative glycolysis, most of which provides ATP to support K^+^ reuptake. However, only approximately half of the functional component of glucose metabolism in the awake non-activated state can be displaced (Chowdhury et al., 2014; Rothman, Behar, & Dienel, 2022). A potential explanation is that glycolytic ATP is required for other processes coupled to the rate of the Glu/GABA/Gln cycle such as packaging glutamate in synaptic vesicles (Takeda & Ueda, 2017; Ueda, 2016). However, at present, there are no studies in vivo directly testing this and other potential relationships.

Each of these topics involves important aspects of excitatory/inhibitory neurotransmitter cycling, and all are necessary to more fully understand the energetics of Glu/GABA signaling.

### 2.2 | When and where is glutamate being used to fuel brain energy metabolism?

The energetic demands of the mammalian brain approach 20% of basal metabolic rate. Up to 60%–80% of this energy is used to support neuronal signaling, and most signaling is mediated by the excitatory neurotransmitter, glutamate, or the inhibitory neurotransmitter, γ-aminobutyric acid (GABA; Attwell & Laughlin, 2001; Buzsáki et al., 2007). Although the brain can utilize other sources of fuels, in vivo studies demonstrate that energetic demands are primarily met by glycolysis and oxidative phosphorylation (Buzsáki et al., 2007; Dienel & Rothman, 2020). Some of this glucose comes from glycogen, while much of it comes from the circulation which also carries O_2_ required for oxidative phosphorylation. While the brain clearly depends on glucose for the bulk of brain energy production, there are alternate sources of fuel, such as glycogen, ketones/fats, and glutamate.

The levels of glutamate in the brain are fivefold higher than those of glucose and approach 10 mmol/Kg. Glutamate is one metabolic step away from the tricarboxylic acid cycle intermediate, α-ketoglutarate, and can generate up to 20 molecules of ATP, depending on the extent to which it is oxidized (Dienel, 2013). In addition to glutamate dehydrogenase, there are transaminases in cytosol and mitochondria that convert glutamate to α-ketoglutarate (Hertz & Rothman, 2017; McKenna, 2013). The abundance of glutamate, combined with a high potential yield of ATP, make it a particularly attractive source of fuel. The glutamate carbon backbone is not exported from the brain, and replenishing this backbone is dependent upon the astrocyte-specific enzyme pyruvate carboxylase ((Sonnewald & Rae, 2010), also see section on pyruvate carboxylase in this article). This makes it possible to monitor the oxidation of glutamate in vivo using an appropriately labeled glucose precursor. Under these conditions, the rate of pyruvate carboxylation is between 13% and 19% of the rate of oxidative glucose consumption (McNair et al., 2022; Rothman et al., 2011). These and other similar observations demonstrate that glutamate is being used as a source of fuel in total brain tissue (Dienel, 2013; McKenna, 2013).

Co-compartmentalization and/or assembly of multienzyme complexes improves the efficiency of flux through multistep reactions by limiting the distance substrates need to diffuse between different enzymatic steps. This is particularly important for the brain with neurons that have specialized compartments that can be up to a meter apart. Even astrocytes have long processes that extend more than 50 μm in rodents and farther in humans (Vasile et al., 2017). Every step of neurotransmission is directly or indirectly dependent upon ATP, including packaging of the neurotransmitter into vesicles, cycling these vesicles on and off the plasma membrane, clearing these transmitters with Na^+^-dependent transporters, and repolarizing the neuronal membrane after opening of ligand- and voltage-dependent ion channels (Attwell & Laughlin, 2001; Harris et al., 2012). This raises the possibility that different steps in synaptic signaling derive energy from different sources of fuel. In fact, high-resolution imaging studies demonstrate that both glycolysis and oxidative phosphorylation are critical for supplying the ATP that fuels specific steps in synaptic vesicle filling and release (Pulido & Ryan, 2021; Rangaraju et al., 2014). Furthermore, the mitochondria, the richest source of ATP, are positioned in the pre- and post-synaptic nerve terminal to both provide a local source of ATP but also to shape Ca^2+^ signaling and vesicle fusion (Ashrafi et al., 2020; Smith et al., 2016; Vos et al., 2010). In *C. elegans*, glycolytic enzymes are assembled into multiprotein complexes in presynaptic nerve termini and support nerve vesicle recycling (Jang et al., 2016). Together, these studies indicate that glycolysis and oxidative phosphorylation support presynaptic activity when glucose is readily available.

Unlike other classical neurotransmitters, most synaptic glutamate is cleared into astrocytes. It is mediated by two transporters called glutamate transporter-1 (GLT-1) and GLutamate ASpartate Transporter (GLAST; Danbolt, 2001). In fact, synaptic activity causes astrocyte membrane depolarization driven by the co-transport of Na^+^ that occurs with each cycle (Bergles & Jahr, 1997). There are small levels of GLT-1 on the presynaptic nerve terminal (Petr et al., 2015), and another member of this family, EAAC1, is also found on neuronal cell bodies and processes (Holmseth et al., 2012). In astrocytes, at least some of this glutamate is converted to glutamine, which is then transported into the extracellular space for subsequent uptake into neurons and reconversion back to glutamine (Dienel, 2013). Several groups have demonstrated that between 10 and 50% of externally provided glutamate is oxidized in astrocytes (McKenna, 2013; McKenna et al., 1996; Yu et al., 1982). There is also evidence that increasing extracellular glutamate increases the percentage of glutamate that is oxidized, but this may be related to glutamate receptor activation rather than substrate-induced activation (McKenna, 2013; McKenna et al., 1996; Skowrońska & Albrecht, 2012). Glutamate transporters are enriched on astrocyte processes, near synapses (Chaudhry et al., 1995; Lehre et al., 1995). Older electron microscopic analyses documented the presence of mitochondria in astrocyte processes (for review, see (Robinson & Jackson, 2016)), and several recent studies demonstrate that mitochondria are found throughout the larger astrocyte processes and branches (Aboufares El Alaoui et al., 2021; Agarwal et al., 2017; Jackson et al., 2014; Jackson & Robinson, 2018; Kacerovsky & Murai, 2016). Both GLT-1 and GLAST anatomically co-compartmentalize with mitochondria and form immunoprecipitable complexes with several glycolytic/mitochondrial proteins (Bauer et al., 2012; Genda et al., 2011). These complexes are found near synapses where synaptic release of glutamate can transiently increase extracellular glutamate to millimolar concentrations (Jackson et al., 2014; Stephen et al., 2015). This co-compartmentalization is consistent with several studies that have demonstrated that both glycolysis and oxidative phosphorylation support glutamate uptake in both astrocytes and brain homogenates enriched in synaptic termini (Balcar & Johnston, 1972; Logan & Snyder, 1972; Roberts & Watkins, 1975; Schousboe et al., 2011; Swanson & Benington, 1996) and with evidence that glutamate uptake is coupled to increased glycolysis and glucose transport (Bittner et al., 2011; Debernardi et al., 1999; Pellerin & Magistretti, 1994; Takahashi et al., 1995) but see (Robinson & Jackson, 2016) for references to studies that do not observe this coupling).

This co-compartmentalization would also seemingly serve to deliver extracellular glutamate directly to mitochondria for oxidation, providing ATP to fuel the Na^+^/K^+^ ATPase that restores the membrane potential. To test this possibility, the effects of glutamate dehydrogenase inhibitors on glutamate uptake were examined in crude P2 preparations that contain synaptosomes, myelin, mitochondria, and glial elements (Whitelaw & Robinson, 2013). In this preparation, three different inhibitors of glutamate dehydrogenase reduce Na^+^-dependent glutamate uptake in a concentration-dependent mannerr. This effect is non-competitive, consistent with an effect on the energy supply required to fuel glutamate uptake. Somewhat surprisingly, these inhibitors also reduced Na^+^-dependent D-aspartate and Na^+^-dependent GABA uptake. As neither D-aspartate nor GABA are substrates for glutamate dehydrogenase, these studies indicate that glutamate dehydrogenase provides energetic support, but that endogenous glutamate is being oxidized. In these preparations, essentially all glutamate uptake is mediated by GLT-1 (Robinson & Dowd, 1996), but selective deletion of GLT-1 from neurons versus astrocytes reveals that more than 85% of the uptake is mediated by neuronal pools of GLT-1, not astrocytic pools of GLT-1 (Petr et al., 2015). This raises the possibility that neuronal glutamate is being oxidized to support uptake into synaptic nerve terminals. An inhibitor of glutamate dehydrogenase also reduces glutamate uptake in cultured astrocytes in parallel with inhibition of glutamate oxidation (Bauer et al., 2012). This is consistent with the notion that extracellular glutamate is fuelling its own uptake, but it is not clear if the glutamate entering via the transporter is fuelling its own uptake or if endogenous glutamate is being used for fuel.

The effects of brain selective deletion of glutamate dehydrogenase have also been examined (Frigerio et al., 2012). While glutamate oxidation is reduced to about 15% of control in astrocytes prepared from these animals, there was no effect on synaptic transmission or synaptic plasticity. If glutamate transport is impaired in these animals, one would have expected altered synaptic plasticity (Barnes et al., 2020), but glutamate-evoked increases in astrocytic ATP are eliminated in astrocytes prepared from these mice (Karaca et al., 2015). Therefore, although there is some evidence that glutamate oxidation may support glutamate clearance, there is a clear need to determine the conditions under which this occurs.

The selection of the fuel is, at least in part, dependent upon the relative concentrations of the substrate that will generally dictate flux through a particular pathway. Glucose is distributed by facilitative transporters. Therefore, although glucose concentrations change dynamically at both a subcellular level and in bulk tissue in vivo (Díaz-García et al., 2019; Roussel et al., 2019), glucose gradients are not imposed by transporters. The levels of glutamate are 5–10 times higher than those of glucose, but glutamate is not uniformly distributed. The highest concentrations of glutamate are in neuronal synaptic vesicles (Andersen et al., 2021; Burger et al., 1989; Patel et al., 2017). It has been suggested that cytosolic pools of glutamate are higher in neurons than in the cytosol of astrocytes because of the high levels and selective expression of glutamine synthetase in astrocytes, but this has not been directly addressed (Rossi et al., 2000). Under resting conditions, extracellular glutamate is ca. 25 nM, and it only transiently increases to mM concentrations with synaptic activity (Herman & Jahr, 2007). The availability of genetically encoded fluorescent sensors that rapidly respond to glutamate over a wide dynamic range from μM to mM should make it possible to monitor glutamate levels and fluxes in various subcellular compartments (Armbruster et al., 2020; Marvin et al., 2018; Rose et al., 2018). Combining these approaches with measurement of ATP or glucose should permit a better understanding of how important metabolites change during different aspects of brain signaling (Barros et al., 2018; Köhler et al., 2020).

Oxidized glutamate is replenished from glucose in a process requiring ATP using pyruvate carboxylase ((Dienel, 2013), see section on pyruvate carboxylase). Therefore, consumption of glutamate imposes a metabolic cost, and one might expect less glutamate oxidation in the presence of glucose. In fact, the opposite is observed in astrocyte cultures, millimolar glutamate decreases oxidation of several different fuels, including glucose. The converse is not true; increasing glucose does not decrease glutamate oxidation (McKenna, 2012). Similar effects of glutamate on glucose oxidation have also been observed in hippocampal slices (Torres et al., 2013). These studies need to be extended to more physiologic conditions where extracellular glutamate only transiently increases to mM concentrations (Flanagan et al., 2018).

In summary, the following key issues need to be further addressed

1. *What is the relationship between glutamate oxidation and neuronal activity under activated conditions* in vivo? As described below, the activity dependence of pyruvate carboxylase does not appear to hold during brain activation. However, estimates of total glial energetics during activation are consistent with glutamate (and GABA) oxidation continuing, but without total oxidation which requires anaplerosis (Rothman, Dienel, et al., 2022). There are several potential explanations for this paradox, but targeted in vivo studies are needed to understand the dependence of the pathway and flux of glutamate oxidation under different conditions. There is also evidence that glutamate is oxidized in reduced preparations, but our current understanding of the conditions under which glutamate is oxidized is somewhat limited.
2. *What are the relative contributions of GDH and AAT to glutamate oxidation and glutamate resynthesis*? In addition, the relative contributions of glutamate dehydrogenase and the amino transferases to the generation of α-ketoglutarate is still a topic of discussion (Hertz & Rothman, 2017; Karaca et al., 2015; McKenna et al., 2016). The field needs better tools to define their contributions to brain energy production. Glutamate dehydrogenase and pyruvate carboxylase are both subject to allosteric regulation by pH, ATP, ADP, and other factors (Bailey et al., 1982; Li et al., 2011) see section on pyruvate carboxylase). Each cycle of glutamate uptake is accompanied by the movement of a proton and transient acidification of cytosol/mitochondria; these raise interesting possibilities of local regulation of glutamate dehydrogenase and pyruvate carboxylase in astrocyte processes that have not been examined (Lambert et al., 2014; Zerangue & Kavanaugh, 1996).
3. *What is* the *role of glutamate oxidation in the pathogenesis and recovery from ischemic brain damage and seizure*? While extracellular glutamate is normally tightly controlled, it increases and remains elevated for prolonged periods of time after a stroke or cardiac arrest (Dávalos et al., 1997; Sandroni et al., 2021). It will be important to learn how this shifts the dependence of brain activity on glutamate oxidation. In addition, intractable epilepsy leads to chronic elevation of extracellular glutamate, which contributes to the progression of the disease. The role of impaired glutamate oxidation in this process is not known. Knowledge in both of these areas could also lead to glutamate oxidation as a target for treatment.
4. *What is the function of the second isoform of glutamate dehydrogenase in primates*? Finally, a relatively recent evolutionary event introduced a second isoform of glutamate dehydrogenase into the genome with expression restricted to apes and humans (Shashidharan & Plaitakis, 2014). This second isoform is expressed in both neurons and astrocytes and displays interesting differences in the Km for glutamate that would favor glutamate oxidation (see (Bunik et al., 2016)). Given the relative enrichment of this isoform in brain tissue, it suggests that brain glutamate has evolved to become even more important as a source of fuel, but this has not been addressed. Regardless of the oxidation of exogenous glucose as fuel, the glutamate consumed must be replenished, and during severe hypoglycemia, metabolism of endogenous brain compounds does not provide sufficient energy to maintain consciousness, that is only restored by provision of glucose.

### 2.3 | When does brain use the anaplerotic enzyme pyruvate carboxylase and under what conditions?

The carboxylation of pyruvate to form oxaloacetate replenishes the 4-carbon pools of the Krebs cycle, which is essential for biosynthetic pathways, such as gluconeogenesis, lipogenesis, and synthesis of neurotransmitters. The reaction (Equation 1) is catalyzed by the mitochondrial enzyme pyruvate carboxylase that is biotin-dependent, requires the acetyl-CoA as an allosteric activator, and hydrolyzes ATP to ADP (Adina-Zada et al., 2012; Jitrapakdee et al., 2008).

(1) $$\text{Pyruvate}+{{\text{HCO}}_{3}}^{-}+\text{ATP}\to \text{oxaloacetate}+\text{ADP}+{\text{P}}_{\text{i}}$$

In 1973, Patel and Tilghman demonstrated that isolated rat brain mitochondria fix HCO_3_^−^ in the presence of pyruvate, ATP, in-organic phosphate, and magnesium. This reaction was inhibited by dissipating the mitochondrial H^+^ gradient with dinitrophenol, and by ADP, malate, succinate, fumarate, and oxaloacetate, but stimulated by glutamate (negligible effect of adding aspartate; Patel & Tilghman, 1973). Pyruvate carboxylase is present in glial cells, especially abundant in astrocytes, while negligibly expressed in neurons (Cesar & Hamprecht, 1995; Murin et al., 2009; Shank et al., 1985; Waagepetersen, Qu, et al., 2001; for controversy discussion, see (Sonnewald & Rae, 2010)).

**FIGURE 2:**
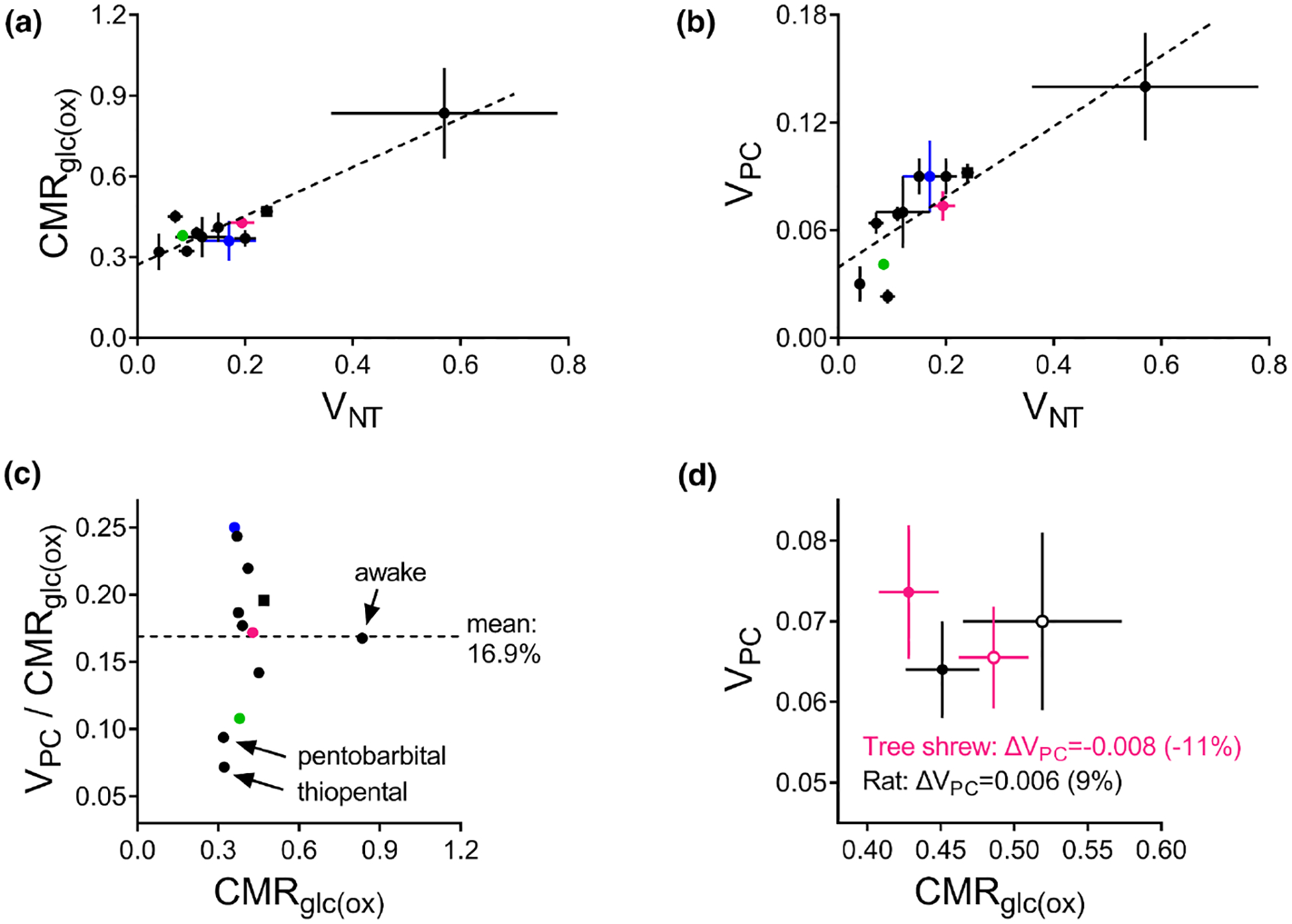
Relations between (a) cerebral metabolic rate of oxidative glucose consumption (CMR_glc(ox)_) and (b) flux through pyruvate carboxylase (*V*_PC_) and through the glutamate/GABA-glutamine neurotransmitter cycle (*V*_NT_). Data are from studies using ^13^C MRS and similar compartmentalized mathematical modelling in the brain of rats (back symbols), mice (green), tree shrews (magenta), or humans (blue). Two experiments on rats were conducted at near-isoelectricity induced by pentobarbital (Choi et al., 2002) or thiopental (Sonnay et al., 2017). One experiment was conducted in awake rats (Öz et al., 2004). (c) *V*_PC_ is on average 16.8% of CMR_glc(ox)_. (d) Acute cortical stimulation results in increased CMR_glc(ox)_ but negligible *V*_PC_ changes (rest = closed symbols; stimulation = open symbols). Circles and squares represent fluxes estimated with a 2-compartment or 3-compartment model, respectively. Metabolic fluxes are shown in μmol/g/min with associated SD (see Table 1).

Magnetic resonance spectroscopy coupled to ^13^C tracing approaches is an excellent tool for studying metabolic pathways and provided evidence for significantly active pyruvate carboxylation in the living brain of rodents (Hassel et al., 1995; Shank et al., 1993) and humans (Gruetter et al., 2001; Mason et al., 2007). Magnetic resonance spectroscopy studies of neuroenergetics have been conducted in vivo over the last three decades and have established a linear relationship between the cerebral metabolic rate of oxidative glucose consumption (CMR_glc(ox)_) and the glutamate/GABA-glutamine neurotransmitter cycle (*V*_NT_), across a wide range of functional states (Hyder et al., 2013; Rothman, Dienel, et al., 2022). Moreover, a flux through pyruvate carboxylation (*V*_PC_) can be determined in such ^13^C tracing experiments by observing the relative labelling of carbons 2 and 3 within glutamate and glutamine molecules. With sensitivity and spectral resolution improvements at high magnetic field, magnetic resonance spectroscopy in vivo during infusion of ^13^C-labeled glucose allowed to observe time courses for all aliphatic carbons of glutamate, glutamine, and aspartate and thus increase the precision in the estimation of metabolic fluxes in astrocytes, including *V*_PC_, e.g., (Duarte et al., 2011; Gruetter et al., 2001; Sonnay et al., 2016). At high magnetic field, not only CMR_glc(ox)_ was linearly related to V_NT_ (Hyder et al., 2013; Rothman, Behar, & Dienel, 2022) but also V_PC_ was increased with V_NT_ across mammal species and anesthesia states (Figure 2a–c).

The regulation of pyruvate carboxylation is complex in the cellular context. While glutamate stimulated pyruvate carboxylation in isolated mitochondria activity (Patel & Tilghman, 1973), it was found to inhibit it in cerebellar astrocytes in vitro (Qu et al., 2001). This could be due to oxidation of glutamate that replenishes Krebs cycle intermediates (McKenna, 2013; Sonnewald, 2014), which in turn inhibit pyruvate carboxylation (Patel & Tilghman, 1973). Thus, glutamate is unlikely to couple *V*_NT_ to *V*_PC_ in the cellular context. On the other hand, in cultured astrocytes, K^+^ elevation stimulates CO_2_ fixation (Kaufman & Driscoll, 1992), which could explain why *V*_PC_ increases with increased brain activity.

In contrast, acute stimulation of cortical activity has led to increased CMR_glc(ox)_ and *V*_NT_, but not *V*_PC_. *V*_PC_ was reported unaltered during somatosensory stimulation of the rat cortex under α-chloralose (Sonnay et al., 2016) and during visual stimulation in the tree shrew cortex under light isoflurane anesthesia (Sonnay et al., 2017; Figure 2d). Absence of V_PC_ stimulation was also observed during bicuculline-induced seizures in rats under halothane anesthesia (Patel et al., 2005) and upon depolarization of brain slices in vitro (Taylor et al., 1996).

In line with absence of pyruvate carboxylase stimulation, Mangia et al. reported glutamate accumulation of approximately 0.2 μmol/g paralleled by equimolar reduction of aspartate concentration in the human visual cortex during acute stimulation (Mangia et al., 2007). It is thus plausible that, in certain conditions, transamination of aspartate could supply oxaloacetate in the absence of activity-stimulated pyruvate carboxylase. In tree shrews under light isoflurane anesthesia, visual stimulation of the cortex did not increase *V*_PC_ nor changed concentrations of glutamate and aspartate (Sonnay et al., 2017). In this particular animal model and stimulation paradigm, glutamate oxidation followed by pyruvate recycling (McKenna, 2013) or by lactate production and eventual release from the activated area (Sonnewald, 2014) could be predominant over pyruvate carboxylation. Furthermore, this tree shrew experiment revealed an activity-induced decrease in the phosphocreatine-to-creatine ratio, suggesting reduced energy charge of the activated cortex (Sonnay et al., 2017). This would be accompanied by a transient increase of ADP that is also capable of inhibiting pyruvate carboxylase activity (Patel & Tilghman, 1973), thus facilitating astrocytic oxidation of glutamate released by neurons rather than ATP-dependent glutamate amidation by glutamine synthetase.

This paradox remains hitherto unexplained,and warrants further research. It is known that pyruvate carboxylase activity is transcriptionally regulated (Jitrapakdee et al., 2008), which does not occur significantly in the time frame of the ^13^C tracing experiments with acute cortical stimulation. Thus, it is likely that cell signaling regulation of energy metabolism genes in astrocytes plays a role in the control of *V*_PC_ during the transition from acute cerebral activation to a persistent high activity state. The signals regulating pyruvate carboxylase expression in the brain remain unknown.

In summary, the following key questions need to be addressed

1. What regulates the expression and translation of pyruvate carboxylase in brain cells?
2. How is the balance between pyruvate carboxylation and amino acid oxidation regulated for maintaining levels of Krebs cycle intermediates in astrocytes?
3. Which signals resulting from synaptic activity effectively regulate pyruvate carboxylation in astrocytes?
4. How is pyruvate carboxylase post-transcriptionally regulated in the brain?

## 3 | BRANCH PATHWAYS TO AND FROM THE KREBS CYCLE: TRAFFICKING AND COMPARTMENTATION

### 3.1 | What are the functions of lactate?

There has been a wealth of papers over the past few decades on lactate metabolism and the role(s) of lactate in the brain; e.g., (Bak et al., 2009; Dienel, 2012; Hollnagel et al., 2020; Kann, 2023; Lauritzen et al., 2013; Magistretti & Allaman, 2018; Mangia et al., 2009), with some 16 000 papers resulting from a search for lactate metabolism AND brain in PubMed. Despite all this investigation, lactate is likely the most controversial and misunderstood brain energy substrate.

**FIGURE 3:**
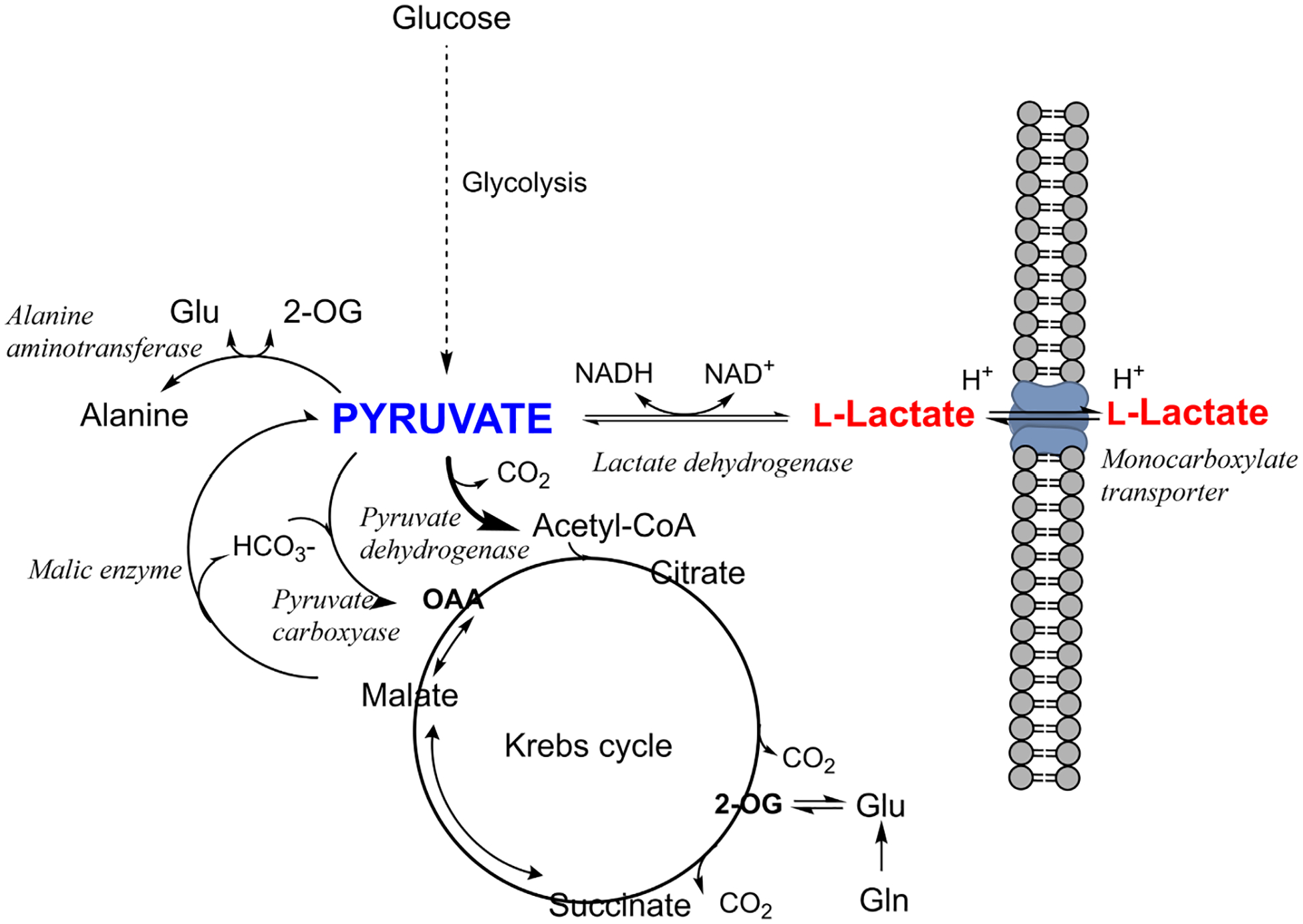
Relationship of lactate to the key metabolic crossroad occupied by pyruvate. Pyruvate is involved in several different reactions, some of which are far-equilibrium reactions with high flux control coefficients, while lactate is in fast exchange with pyruvate and the external milieu. Lactate therefore reflects the availability of its source substrate, pyruvate. OAA, oxaloacetate; 2-OG, 2-oxoglutarate (α-ketoglutarate).

Lactate is formed in all brain cells solely from pyruvate in a reversible reaction catalyzed by lactate dehydrogenase, requiring NADH as the cofactor. Lactate dehydrogenase is a ubiquitous near-equilibrium enzyme, which means it has limited capacity to influence metabolic control (Fell, 1997). While lactate itself is a “dead-end” metabolite, its immediate substrate, pyruvate, is a key molecule at a metabolic cross-road (Figure 3). Pyruvate can be generated by oxidative metabolism of glucose, glutamate, and other substrates via the pyruvate recycling pathway (McKenna et al., 1996; Olsen & Sonnewald, 2015; Sonnewald, 2014). It is converted to acetyl-CoA via the far-equilibrium pyruvate dehydrogenase complex and can also be transaminated to alanine in a pairing with glutamate and 2-oxoglutarate or carboxylated to form oxaloacetate by pyruvate carboxylase, a glial-specific enzyme (McKenna et al., 2012). This plethora of possible pathways for pyruvate (Figure 3), and their respective thermodynamics, means that pyruvate clearance rates (i.e., substrate availability) tend to have more control over production of lactate than lactate dehydrogenase itself (Bak & Schousboe, 2017; Nortley & Attwell, 2017). Lactate isoenzymes, while able to influence the rate (timecourse) of conversion of substrates, have no influence on the equilibrium lactate concentration (Quistorff & Grunnet, 2011).

Another important, and often forgotten factor, is that full lactate utilization in the respiratory chain requires oxygen (CMRO_2_), and if oxygen consumption does not match glucose utilization, then the lactate derived from glucose cannot be oxidized. During activation, glycolysis is preferentially upregulated compared with oxidation (Dienel & Cruz, 2016). Specific activity measurements indicate that most of the lactate produced from glucose via this upregulation is retained and oxidized in the brain (Dienel & Cruz, 2009). Specific activity measurements also indicate that lactate derived from glycogen in astrocytes is quickly released from the brain because, if retained, lactate-specific activity would be diluted (Dienel & Rothman, 2019).

Lactate may escape from its “dead-end” reaction by leaving the cell, which it does through an array of transporters which mediate the uptake and release of lactate and other monocarboxylates such as acetate, pyruvate, and the ketone bodies β-hydroxybutyrate and acetoacetate (Rae et al., 2012), Lactate may also escape via gap junctions where it travels relatively long distances (Dienel, 2017a).

It is important to note that MCT transporters cotransport a proton (H+) with each monocarboxylate, meaning that they are pH-sensitive. They are passive transporters which therefore exert minimal control over the direction of lactate fluxes; in practice, they are subject to trans-acceleration (Deuticke, 1982), meaning that exchange reactions (one monocarboxylate is swapped for another) occur more rapidly than unidirectional ones (Poole & Halestrap, 1993). They therefore play a role facilitating the rapid exchange of monocarboxylates between compartments in the brain.

#### 3.1.1 | What is the fate of lactate in the brain?

Lactate production in the human brain appears to be region-specific in a pattern that is conserved across individuals, with production highest in the parietal and occipital lobes (cuneus, precuneus, cingulate, and lingual gyri). Production of lactate is also not necessarily related to regional tendency to aerobic glycolysis or FDG uptake (Lee et al., 2020), although it may be related to the availability of NADH. Exactly when and why the brain produces lactate is still not well-understood, although many possible explanations have been advanced. It has been speculated that the need to rapidly clear glutamate from the brain is fueled by glycolysis, producing pyruvate which is excess to requirements. The excess pyruvate is then effluxed as lactate, regenerating cytosolic NAD^+^ (Shulman et al., 2001).

Alternatively, the controversial astrocyte–lactate shuttle hypothesis posits that lactate is released by astrocytes in response to glutamate release from neurons, where the lactate is taken up and used as a fuel. Many groups have embraced the concept of an astrocyte to neuron lactate shuttle (ANLSH/ANLS) since it was first proposed by Pellerin and Magistretti based on in vitro studies (Pellerin & Magistretti, 1994). However, studies in vivo supporting this shuttle are few and not convincing [reviewed in (Díaz-García et al., 2017; Díaz-García & Yellen, 2019; Dienel, 2017b, 2019; Yellen, 2018).

The thermodynamics of lactate metabolism, transport, and clearance go some way to explaining the attraction of the ANLS. As the reactions involving lactate are all near-equilibrium enzymes, lactate is highly correlated with many metabolites and processes, which gives lactate a seeming importance beyond the reality. This was explained succinctly by the renowned biochemist, Richard Veech, who stated that “measurements of lactate content per se are able to provide relatively little information other than the level of lactate itself. By combining the measurement of lactate with the concentration of its relevant metabolic partners, a great deal more useful information can be gained about the state of the tissue” (Veech, 1991).

So is there an astrocyte–neuron lactate shuttle? Sometimes, under the right conditions but it is probably just lactate exchanging between compartments. Is it important? Well, it can happen, but in the grand scheme of things, no, it is not that important, as lactate is just as likely to efflux from neurons and is not a substrate that can maintain high level neuronal function (Bak et al., 2006, 2009; Kann, 2023; York et al., 2023). Giving it a name (ANLS) has given it an importance, which outstrips its role as one of many reasons that lactate enters or leaves cells and the brain.

A recent modeling study posited that the production of lactate and its subsequent clearance from the brain was a mechanism for maintaining brain homeostasis; i.e., clearance of excess protons to keep pH, pCO_2_, and pO_2_ stable (DiNuzzo et al., 2023). Certainly, build-up of lactate can have deleterious effects (Griffin et al., 1999; Nedergaard et al., 1991). This model also helps explain the purport of the large positive blood oxygen level-dependent (BOLD) response (the mainstay of brain functional magnetic resonance imaging (Gauthier & Fan, 2019)) where delivery of blood flow and oxygen far in excess of requirements can be seen after brain activation.

Other explanations for the excess production of lactate include a role for lactate as a volume transmitter (Bergersen & Gjedde, 2012), whereby lactate acts as a messenger in the distribution of cellular signals and thereby maintains the metabolic network via two main mechanisms. 1. By regulating the formation of cAMP via the lactate receptor GPR81 (see below) and/or 2, by adjusting the NADH/NAD^+^ redox ratios. This latter role would seem unlikely given recent work showing that lactate import did not contribute to, nor was necessary for, neuronal cytosolic NADH transients on activation (Díaz-García et al., 2017).

Lactate thermodynamics tell us that lactate is a molecule that exchanges rapidly between the different compartments in the brain, including rapidly moving relatively long distances via gap junction connexins (Dienel, 2017a; Giaume et al., 1997). There is evidence that lactate diffuses out of the brain, for example, via perivascular spaces (Ball et al., 2010) and that this may occur more rapidly in sleep (Benveniste et al., 2019). Experiments examining these brain drainage systems are complex to undertake, particularly in vivo and the results are variable (Barisano et al., 2022).

#### 3.1.2 | The role(s) and importance of the lactate receptor are not well-understood

The lactate receptor GRP81 (HCAR1) is enriched on the blood–brain barrier, concentrated on the postsynaptic membranes of excitatory synapses in the cortex and hippocampus (Bozzo et al., 2013). Activation of the receptor by lactate or the agonist 3,5-dihydroxybenzoate reduces cAMP (Lauritzen et al., 2013) and decreases spontaneous Ca^2+^ spiking in neurons (Bozzo et al., 2013). Although localization of the receptor is thought to indicate a role for lactate as a volume transmitter in brain signaling, the role(s) are not well-understood. Studies by the Chatton group concluded that “HCAR1 activation in neurons causes a downmodulation of neuronal activity through presynaptic mechanisms and by reducing neuronal excitability” (Briquet et al., 2022; de Castro Abrantes et al., 2019). They reported functional cross-talk of the receptor with other GPCRs “for the fine tuning of neuronal activity” (de Castro Abrantes et al., 2019). Other studies suggest that “L-Lactate acts as a signalling molecule for neuroprotection against excitotoxicity through coordinated cellular pathways involving ATP production, release and activation of a P2Y/KATP cascade” (Jourdain et al., 2016). Recent studies indicate a role of the GRP81 in developmental brain angiogenesis, neurovisual development, and recovery from ischemic injury in the developing brain (Chaudhari et al., 2022; Laroche et al., 2021). In contrast, other studies showed that the inhibition of GPR81 protected brain cells from ischemic injury (Shen et al., 2015). It has also been suggested that GPR81 mediates central fatigue after exercise (Li et al., 2022), which could make the receptor a possible target for intervention in fatigue-related disorders such as myalgic encephalomyelitis.

#### 3.1.3 | What is the role of lactate in oligodendrocytes?

Studies from several groups show that lactate released by oligodendroglia supports metabolism in axons (Nave et al., 2023; Philips & Rothstein, 2017). Lactate release from the breakdown of glycogen can provide energy to axons during hypoglycemia (Brown & Ransom, 2007). The localization of MCT1 on the innermost myelin layer that faces the periaxonal space and MCT2 on axons facilitates the trafficking of lactate or pyruvate to axons (Nave et al., 2023). There is evidence that oligodendrocytes also provide glucose to axons (Fünfschilling et al., 2012; Meyer et al., 2018). Oligodendrocyte metabolism is a relatively unexplored field (Amaral et al., 2016) which has largely been ignored in metabolic modeling. It remains to be seen if oligodendrocyte metabolism of lactate is different to that in neurons and other glial cells as more data are needed.

In summary, systems-level neurochemical investigations, where multiple relevant metabolites are measured in a brain or brain-like model with interacting cell types, are required to better understand the role(s) of lactate. As a molecule which is in exchange and equilibrium with many brain compartments, study of lactate has to be in the context of all the neighboring reactions.

### 3.2 | Why is the sodium-dependent citrate transporter SLC13A5 important in brain?

It has been known for many years that astrocytes in culture, and likely in the brain in vivo, release citrate (Westergaard et al., 1994) which may act as a chelator to modulate Zn^2+^-mediated inhibition of NMDA-receptor-mediated glutamate release (Westergaard et al., 1995, 2017). Citrate also chelates Mg^2+^, which could potentially control the amount of Mg-ATP (Badar-Goffer et al., 1990), and when released citrate may act to buffer Ca^2+^ and Mg^2+^ in the extracellular milieu (Westergaard et al., 1994). Early NMR studies revealed high enrichment of citrate from metabolism of acetate and from glucose metabolism under depolarizing conditions (Badar-Goffer et al., 1990, 1992). Under resting conditions, the intracellular concentration of citrate is similar in neurons and astrocytes (Westergaard et al., 1994). Citrate released from the mitochondria into the cytosol of cells can be cleaved by ATP citrate lyase to oxaloacetate, which can be converted to malate and participate in the malate–aspartate shuttle, and acetyl CoA which can be used for lipid synthesis and/or acetylation of proteins (McKenna et al., 2006; McKenna & Ferreira, 2016; Pougovkina et al., 2014). Cytosolic citrate inhibits phosphofructokinase 1 (PFK1), the key regulatory enzyme of glycolysis (Garland et al., 1963; Passonneau & Lowry, 1963). Astrocytes have a releasable pool of citrate, and the finding that incubation with bicarbonate and/or with K^+^ stimulates this release suggests a role for the enzyme pyruvate carboxylase in formation of the releasable pool (Waagepetersen, Sonnewald, et al., 2001; Westergaard et al., 1994). The extent to which astrocytes release citrate in vivo and the conditions leading to such release are not known.

Exogenous citrate can potentially be used for energy since it is a Krebs cycle intermediate formed in mitochondria by condensation of acetyl CoA with oxaloacetate. As uptake by neurons was thought to be low, citrate has not been considered an important candidate for metabolic trafficking from astrocytes to neurons in the brain. This view is being reevaluated subsequent to the identification of SLC13A5, a sodium-dependent, plasma membrane citrate transporter that is highly expressed in specific neuronal populations (Bhutia et al., 2017). The identification of patients with loss of function mutations in SLC13A5 who have early-onset epilepsy and neurodevelopmental delay (Bhutia et al., 2017) has led to renewed interest in the role(s) of citrate in the brain. The severity of symptoms in human infants with loss of function mutations who have seizures from the first day of life suggests an important role of neuronal uptake of citrate via this transporter in brain function (Henke et al., 2020; Thevenon et al., 2014). Interestingly, the overexpression of SLC13A5 in neurons in mice is associated with alterations in the structure and function of synapses, impaired integrity of white matter, and autistic-like behavior (Rigby et al., 2022).

The question of why the plasma membrane citrate transporter is highly expressed on specific neuronal populations warrants further investigation (Bhutia et al., 2017). There is a clear need for more studies to better understand the role of the plasma membrane citrate transporter SLC13A5 in the brain, whether extracellular citrate is an important energy substrate for neurons, and the extent and regulation of citrate release by astrocytes in vivo.

### 3.3 | Brain acetate metabolism is compartmentalized, but why?

Metabolism of the two-carbon monocarboxylic acid anion acetate in the brain has long been known to be compartmentalized; indeed, this compartmentalization has formed the basis for substrate-specific investigations of glial and neuronal metabolism (Hassel et al., 1995, 1997). The basis for the compartmentalization was first noted by the observation that [^14^C]acetate labels brain glutamine with higher specific radioactivity than glutamate (O’Neal et al., 1966; O’Neal & Koeppe, 1966) and that the relative fractional enrichment of glutamine was much greater than that of glutamate when acetate was supplied as a labeled substrate (Minchin & Beart, 1975; van den Berg et al., 1969). These results indicated that acetate was metabolized significantly more in a compartment that also contained glutamine synthetase; i.e., a glial compartment (Martinez-Hernandez et al., 1977). While the idea of mitochondrial heterogeneity explored in (Salganicoff & de Robertis, 1963) rein-forced the idea of two distinct sets of Krebs cycles in the brain, other explanations for the glutamate–glutamine labelling profile had not yet been excluded. An impactful investigation in the 1990s attributed this glial preference to failure by neurons to take up acetate in any meaningful quantities (Waniewski & Martin, 1998), and for some time it was believed that the neuronal monocarboxylate transporter (MCT2) had a very low affinity for acetate. This was debunked when the affinity of MCT2 for acetate was measured and found to be similar to that of other monocarboxylates (Rae et al., 2012). Neurons were therefore capable of taking up acetate and in fact do, as demonstrated in cultured neurons (Brand et al., 1997), hippocampal nerve terminals (Carroll, 1997), isolated synaptosomes (Berl et al., 1983), and whole brain (Chapa et al., 1999). Neurons also possess a sodium-dependent monocarboxylate transporter (sodium-dependent monocarboxylate transporter 1; SLC5A8) which has a higher affinity for acetate than MCT2 (Babu et al., 2011), although the exact contribution of this sodium-dependent monocarboxylate transporter to neuronal acetate uptake is not known. Further, the uptake of acetate across the blood–brain barrier is not rate-limiting, but rather the rate-limiting factor is the actual metabolism of acetate in the brain (Deelchand et al., 2009; Patel et al., 2010).

This begs the question; why do neurons metabolize less acetate than astrocytes and why is acetate a poor substrate in the brain?

Metabolism of acetate in the brain is limited in ways that the metabolism of other substrates, such as glucose and pyruvate, are not. In the brain slice, supplying acetate above 0.4 mmol/L actually reduces overall metabolism of glucose, with the excess acetate remaining unused (Rowlands et al., 2017). Increasing metabolic demand via potassium depolarization (Rowlands et al., 2017) or through visual stimulation (Dienel & Cruz, 2006) also reduces acetate metabolism, despite the resultant metabolic demand being mostly shouldered by the glial compartment. As acetate is made up of two carbons, it does not alone support the net synthesis of Krebs cycle-derived metabolites as two carbons are lost with every cycle. This may offer one explanation for why an experiment incubating synaptosomes with acetate failed to show significant metabolism, as the need for ATP hydrolysis in the initial step of metabolism suggests why a synaptosomal preparation might have reduced capacity to use this substrate.

It may be that neuronal capacity to metabolize acetate is limited by the expression of acetyl-CoA synthetase (AceCS: E.C. 6.2.1.1), the enzyme responsible for allowing acetate to enter metabolism in an ATP-requiring process. The brain has two isoforms; acetyl-CoA synthetase 1, located mostly in nuclear and within some cytoplasmic compartments (Ariyannur et al., 2010), and acetyl-CoA synthetase 2 which is a mitochondrial matrix enzyme (Fujino et al., 2001). The protein levels of AceCS2 have been reported to be low in the brain and mostly localized to the mitochondria located in astrocytic endfeet (Moffett et al., 2013). Previous inferences about neuronal acetyl-CoA synthetase levels (e.g. were based on its activity) in a synaptosomal fraction vs a whole brain-derived mitochondrial fraction and did not discriminate the enzyme isoform (Tucek, 1967); indeed the author mentions that it is not possible to determine whether citrate or acetate is more important in the synthesis of acetylcholine using the method employed (Tuček, 1967). Reference to the mRNA expression levels in the mouse frontal cortex (http://dropviz.org) indicates that expression of ACCS1 (which encodes acetyl-CoA synthetase 2) is very low in oligodendrocytes (0.693 normalized mean log relative to the expression of other genes in the frontal cortex) and one of the interneurons investigated (0.693) and negligible in other neurons investigated such as in layers 5, 6, and 23 (0.00). This compares with relatively high expression in cortical astrocytes (2.71) of both ACSS2 and ACSS1. Transcripts of the cytosolic enzyme (acetyl-CoA synthetase 1, encoded by ACSS2) by comparison is expressed more strongly in neurons (log values ranging from 1.1 to 1.61) and oligodendrocytes (1.79). It should be noted that the single-cell method used here is vulnerable to impurities, used a relatively immature (p14) mouse brain, and is from the transcriptome, not the proteome (Macosko et al., 2015), although proteome data also indicate that expression of both isoforms is relatively low in neurons (Sharma et al., 2015). These data imply that some neurons, at least, have a very low level of mitochondrial acetyl-CoA synthetase.

Understanding why, under basal conditions, acetate is metabolized primarily in astrocytes may therefore be related to the activity of the acetyl-CoA synthetase proteins within each cell type. The activity of both acetyl-CoA synthetase 1 and acetyl-CoA synthetase 2 is decreased significantly by acetylation (Hallows et al., 2006), with activity restored by deacetylation catalyzed by the silent information regulators SIRT1 and SIRT3, respectively (Hirschey et al., 2011). Although activators of SIRT1 are available and the impact of these on brain metabolism has been investigated (Rowlands et al., 2015, 2017), activation of SIRT1 has multiple metabolic effects (Imai, 2011), making it difficult to isolate the role of acetyl-CoA synthetase activity under these circumstances. Further, acetylation of acetyl-CoA synthetase 2 at K642 in humans (Schwer et al., 2006) or K635 in the murine homolog (Hallows et al., 2006) can, like for acetyl-CoA synthetase 1, completely inactivate the enzyme. However, the direct effects of SIRT3 on acetyl-CoA synthetase 2 activity in brain tissue are currently unknown.

Acetylation of enzymes involved in metabolism as well as histones may offer a “sink” of acetate as well as affecting the rates of metabolic reactions (Wang et al., 2010) and the availability of acetyl-CoA (Shi & Tu, 2015). Deacetylation does not contribute significant amounts of acetyl-CoA to metabolism (Soaita et al., 2023), suggesting that there is regulation or compartmentation of the fates of acetyl-CoA in line with its role as a key molecule in many pathways (Cai & Tu, 2011).

Preferential use of acetate in astrocytes may also be related to the differences in cellular acyl-CoA hydrolase activity (EC: 3.1.2.1). Acetyl-CoA can be converted to acetate by acyl-CoA thioesterase (ACOT). There are 15 characterized ACOTs, of which ACOT1, 2, 5, 7, and 9 are localized to the brain (Ellis et al., 2015; Kirkby et al., 2010; Tillander et al., 2017). In the whole brain sample, acetyl-CoA hydrolase activity is approximately five-fold higher in the intra-mitochondria space (Szutowicz et al., 1982; Szutowicz & Lysiak, 1980). While the majority of thioesterase activity occurs in neurons (Ellis et al., 2013, 2013), it is unknown what proportion of this activity directly relates to acetyl-CoA hydrolysis. It is also currently unknown which ACOTs are responsible for the hydrolysis of Acetyl-CoA to acetate in the brain. ACOT12 is the only cytosolic ACOT with specific hydrolytic activity toward acetyl-CoA (Hunt et al., 2014; Suematsu & Isohashi, 2006). However, as ACOT12 protein or mRNA expression is not detectable in the brain (Ellis et al., 2015; Horibata et al., 2013), the enzyme responsible for acetyl-CoA thioesterase activity within the cytosol remains elusive in the brain. Regarding mitochondrial-specific acetyl-CoA thioesterase activity, a recent paper showed that in the liver tissue of C57BL/6 mice lacking Acot9 expression, ACOT9 regulates the trafficking of short-chain acyl-CoAs (C:2-C:4) at the inner mitochondrial membrane, resulting in a reduction in Krebs cycle metabolites (Steensels et al., 2020). ACOT9 also limits the acetyl-CoA used for mitochondrial lysine acetylation and may play a similar role to that of SIRT3 in reducing overall acetylation within the mitochondria (Steensels et al., 2020). It remains to be seen if ACOT9 can have a similar effect on brain metabolism. However, activity and/or expression differences of ACOT9 in neurons and astrocytes, potentially due to the different metabolic requirements of the cells to regulate lipid metabolism might be a possible explanation for why neurons have a limited capacity to metabolize acetate.

Further support for acetate use being tied to the metabolic needs of the cells comes from studies that have observed increased acetate use to synthesize GABA by GABAergic neurons under conditions of increased SIRT1 activity (Rowlands et al., 2017) or where the contribution from glutamine is removed through inhibition of glutamine synthetase, with about 30% of newly synthesized GABA being derived directly from neuronal metabolism of acetate (Andersen et al., 2017; Rowlands et al., 2017). More recently, it was observed that acetate is preferentially consumed over glucose in a brain-specific Pdha1 knockdown mouse model, where pyruvate dehydrogenase activity in the brain was reduced by 68% (Jakkamsetti et al., 2019). The pyruvate dehydrogenase complex ordinarily plays a principal role in maintaining acetyl-CoA levels within the mitochondrial compartment. In this knockdown model, acetate supplementation stimulated metabolic flux into Krebs cycle intermediates, likely playing a role as an energy substrate in glutamatergic and GABAergic neurons (Jakkamsetti et al., 2019).

**FIGURE 4:**
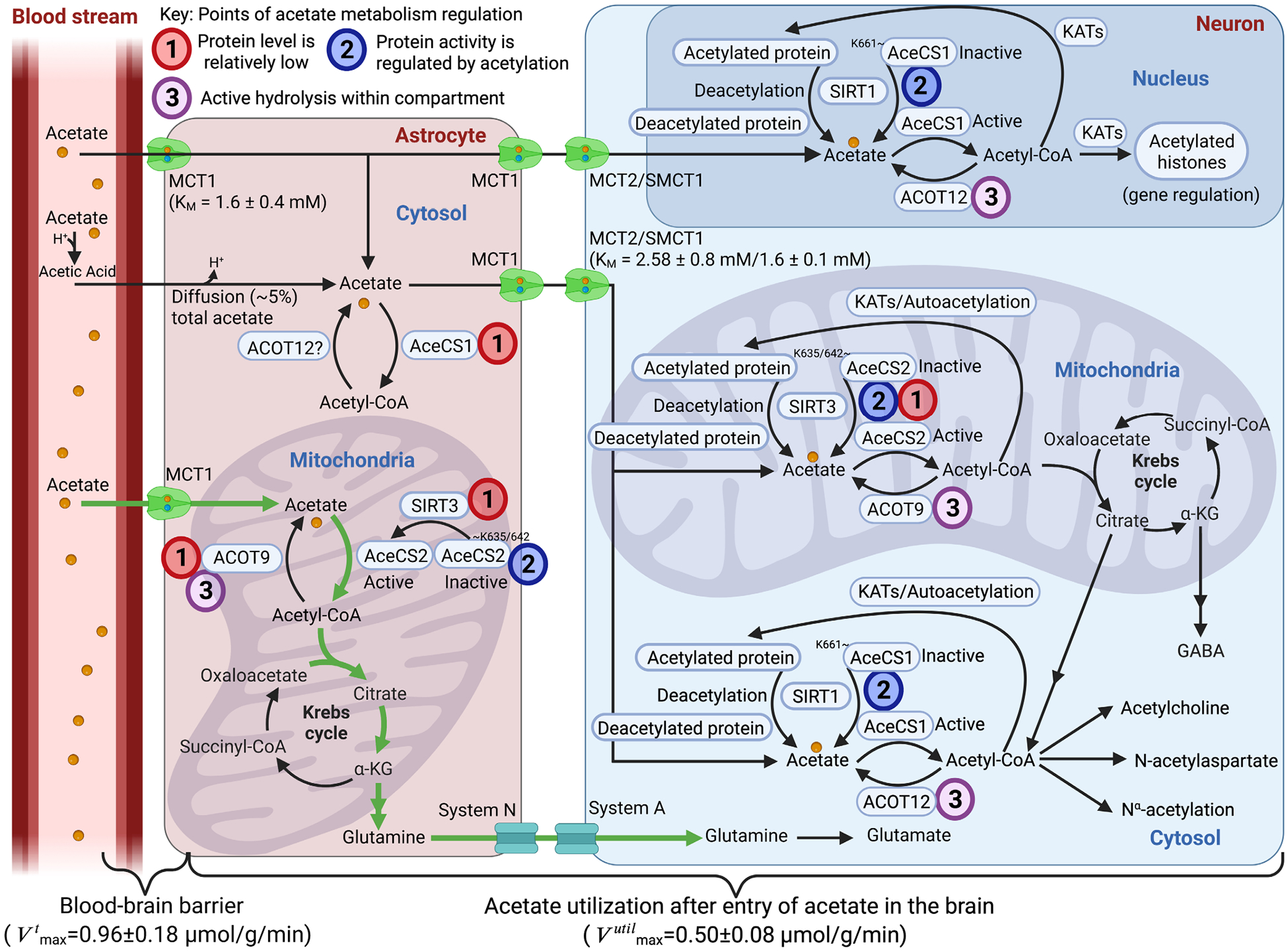
The transport of acetate into the brain exceeds the brain’s capacity to metabolize acetate. Acetate primarily enters the brain through monocarboxylate transporter 1 (MCT1) located in the astrocyte end feet in contact with blood vessels, a small portion of acetate can passively diffuse across the BBB in the form of acetic acid. Intracellular acetate is actively transported into the mitochondrial compartment and converted into acetyl-CoA by acetyl-CoA synthetase 2 (AceCS2; ACSS1, acyl-CoA synthetase short-chain family member 1). Entry of Acetyl-CoA into the astrocytic Krebs cycle can be used to synthesize glutamine which is transported into neurons for glutamate synthesis via the N and A transport systems (green arrows indicate primary use in brain). Regulation of this pathway is thought to be limited as both the protein levels of silent information regulator 3 (SIRT3), which regulates AceCS2 activity, and acyl-CoA thioesterase 9’s (ACOT9) hydrolytic activity is low compared to neurons. Acetate may also be exported through MCT1 located near the surface of neurons that can actively uptake acetate through MCT2/sodium MCT1. Neuronal acetate can be converted to acetyl-CoA in the cytosolic or nuclear compartments by AceCS1 (ACSS2, acyl-CoA synthetase short-chain family member 2), where it can be used by lysine acetyltransferases (KAT)s to acetylate histones for gene regulation, non-histone proteins, and metabolites and play a role in protein synthesis through N^α^-acetylation. Acetyl-CoA may also be used for several biosynthetic pathways including the synthesis of acetylcholine and N-acetylaspartate for use in fatty acid synthesis. Acetate may also be actively transported into the neuronal mitochondrial compartment; however, the ability for neurons to use acetate for acetyl-CoA production may be limited in neurons due to the relatively low protein levels of AceCS2 and the relatively high levels of acetyl-CoA hydrolysis activity (ACOT9). However, as neurons are capable of using acetate under metabolically challenged conditions, neuronal capacity to convert acetate into Krebs cycle intermediates may be linked to the energy status of the cell, with neurons possessing relatively high SIRT3 protein levels, which would play a role in deacetylating and activating AceCS2 within neurons. Silent information regulator 1, SIRT1; acyl-CoA thioesterase 12, ACOT12; α-KG, α-Ketoglutarate; GABA, γ aminobutyric acid.

Taken together, while acetate is *mostly* metabolized in a glial compartment, it is not an *exclusive* marker of glial metabolism as acetate is metabolized in GABAergic neurons. Our present understanding of acetate metabolism is illustrated in Figure 4. Current limitations in labelling experiments prevent us from determining whether acetate is also metabolized in glutamatergic neurons. Expression and regulation of acetyl-CoA synthetase 2 activity via SIRT3 deacetylation and intramitochondrial acetyl-CoA thioesterase activity are promising candidates for explaining why acetate is preferentially incorporated into the glial TCA-cycle.

## 4 | ENERGETICS OF SIGNAL PROCESSING AND HIGHER BRAIN FUNCTIONS

### 4.1 | The demand–supply problem in neuronal bioenergetics

Brains are metabolically vulnerable organs that encompass the largest fuel consumption per unit weight of any organ in the body. The vulnerability is manifest in conditions such as acute hypoglycaemia associated with endocrine pathology, where a sudden loss of fuel is rapidly followed by the onset of neurological symptoms. The vulnerability indicates that neural circuit function, and hence the cellular basis of this function, is itself intolerant to changes in fuel availability.

Pioneering studies from Paul Gold and colleagues (Gold et al., 1986; McNay et al., 2000) showed that the function of certain brain circuits is likely, under normal ‘healthy’ conditions, limited by the availability of an adequate combustible carbon source, and that this local fuel scarcity can impact brain performance. They showed that acutely increasing glucose availability (as opposed to chronic elevations in diabetes) had a profound impact on both working memory (McNay et al., 2000) and inhibitory avoidance (Gold, 1986) tasks. Working memory in humans has also been reported to be significantly enhanced after glucose, but not a non-caloric artificial sweetener, ingestion (Sünram-Lea et al., 2001). Furthermore the impact of increasing glucose availability is more pronounced in aging animals (McNay & Gold, 2001), consistent with the idea that many metabolic processes, including fuel delivery, become compromised with advancing age. Reduced glycolytic metabolism in the brain is additionally considered an early predictor of eventual neurological dysfunction (Mosconi et al., 2008). These findings strongly suggest that aging-related cognitive dysfunction, such as neurodegenerative disease-associated dementia, is closely connected to either loss of metabolic support and/or additional lesions in how possible carbon sources can efficiently be used in neuronal bioenergetics.

As decreased cognitive performance is typically associated with a degradation in synapse function and since nerve terminals can be located at enormous distances on the molecular scale with respect to the cell body, understanding the local bioenergetic rules of nerve terminals will likely hold important clues to eventual therapeutic remedies for cognitive decline. It was previously shown that nerve terminals are intolerant of fuel restriction (Rangaraju et al., 2014), suggesting they may represent one of the loci of the brain’s overall metabolic vulnerability. Minutes after fuel withdrawal, nerve terminals fail to recycle synaptic vesicles, leading to a rapid impairment of synaptic transmission (Ashrafi et al., 2017; Rangaraju et al., 2014). The rapidity of the failure reflects the fact that nerve terminals have a high basal ATP consumption (Pulido & Ryan, 2021) and do not store ATP itself. In this line, neuronal gamma oscillations (30–70 Hz), which require precise synaptic transmission between glutamatergic pyramidal cells and fast-spiking GABAergic interneurons, are highly sensitive to metabolic stress induced by shortage in glucose and oxygen availability ((Elzoheiry et al., 2020; Hollnagel et al., 2020; Kann et al., 2014).

Understanding how brain metabolism is regulated at a molecular level remains a central open question. While tissue bioenergetics is well-established in organs like the muscle, pancreas, liver, and cancer cells, the metabolic demands of neurons and their specific locations are still not well-understood. Neurons are complex in their spatial organization, and the brain is heterogeneous in its cell types and activity, making metabolic tracing techniques difficult to interpret.

The problem of bioenergetics is one of supply and demand, and balancing the two requires upregulation of fuel delivery and oxidation to meet increased demands. Much of what we know about bioenergetics comes from muscle biochemistry and physiology, which also places high demands on metabolic support. However, our understanding of bioenergetics in the brain lags behind.

Although regulating fuel delivery is an essential feature of the hemodynamic response in the cranial vasculature (Iadecola, 2004), stimulation of fuel import and oxidation also occurs in individual neurons (Ashrafi et al., 2017, 2020; Díaz-García et al., 2021). The details of the molecular control of this regulation are still only primitively understood. One of the major gaps in our fundamental understanding of neuronal bioenergetics relates to metabolic flexibility and the molecular pathways that govern this. It is well-appreciated that muscle cells adeptly switch between using glucose, glycogen, and stored triglycerides to fuel the ongoing bioenergetic needs using interesting feedback (Garland et al., 1963; Randle et al., 1963) and hormonal (Hearris et al., 2018) regulation that governs these choices. We know much less about this for neurons, in particular as neither triglycerides nor glycogen are thought to accumulate in neurons in large quantities and thus were considered unlikely to be important to serve as a bioenergetic fuel. At the molecular level, however, important clues have emerged in the last decade suggesting that the scarcity of these ‘fuel depots’ in neurons may reflect only that at steady state they do not build up, but the flux through these pathways may be quite impactful. For example, impairing glycogen metabolism through molecular genetic approaches has a significant impact on cognitive performance in mice (Duran et al., 2013; Duran, Gruart, Varea, et al., 2019) and makes them much less tolerant to metabolic insults (Saez et al., 2014). As glycogen storage and breakdown is a dynamic process, and in tissues outside the brain this is known to be controlled by cAMP, using the presence or absence of this stored carbohydrate as a measure of the relevance to the tissue or cell’s function is misleading. In the future, it will be important to determine when and under what circumstances neurons use different fuels.

Similarly, a critical clue from hereditary spastic paraplegia (HSP) in humans arose in the last decade regarding triglycerides in neurons. Although hereditary spastic paraplegias are typically characterized as “long axon” diseases, as the prominent clinical presentation is limb contractures, a subset of hereditary spastic paraplegias are referred to as complex, as they also present with a strong CNS impairment. Hereditary spastic paraplegia-54 is one such variant and is caused by loss of function of the protein DDHD2 (E.C. 3.1.1; phospholipase DDHD domain-containing protein 2). Through lipidomic analysis of DDHD2 knock-out mice, Inoles and colleagues determined that DDHD2 is a neuron-specific triglyceride lipase (Inloes et al., 2014), and loss of this enzyme’s activity leads to a massive accumulation of triglyceride-containing lipid droplets throughout neurons in the brain, consistent with the fact that hereditary spastic paraplegia-54 patients have large lipid accumulations in their brains (Thabet et al., 2020). DDHD2 KO mice also show significant functional deficits (Inloes et al., 2014), consistent with CNS impairment (intellectual disability and ataxia) that presents in hereditary spastic paraplegia-54 patients (Schuurs-Hoeijmakers et al., 2012). The hereditary spastic paraplegia-54 phenotype in humans and that in the DDHD2 KO mice indicates that rather than never using lipid droplets, they are likely used all the time. If this is so, it brings up the important question of where the fatty acids used to generate lipid droplets originate from. Noteworthily, another enzyme regulating mobilization of fatty acids from lipid droplets, the hormone-sensitive lipase, has been reported to be enriched in synapses (Skoug et al., 2022).

The examples mentioned above point to several critical knowledge gaps we have about the fundamentals of neuronal bioenergetics.

Many cancer cells are known to have profound changes in their metabolism (e.g., the Warburg effect), in part to allow adequate production of all the parts (lipids, nucleotides, amino acids, etc.) necessary to allow rapid cell proliferation. Although neurons are postmitotic, their complex and elaborated architecture dictates that they, too, constantly face the challenge of producing these essential cellular components as part of the simple proteo- and lipid stasis. How this set of demands intersects with the bioenergetic demands associated with neuronal activity is largely terra incognita but will be fertile ground for further discovery.

### 4.2 | How does brain insulin modulate metabolism, cognition, and memory?

Insulin’s effects on brain functioning are widespread. An increase in hypothalamic insulin signals satiety (Baskin et al., 1987; Gerozissis et al., 1997, 1998; Orosco et al., 1995), and postprandial increases in insulin across multiple brain regions (including, e.g., cerebellum, prefrontal cortex, and hippocampus) decrease eating behaviours (Gerozissis et al., 2001; Könner et al., 2011; Kroemer et al., 2013; Kullmann et al., 2015). Insulin also modulates cellular metabolism in some brain regions, including stimulation of cell membrane translocation of glucose transporter 4 (GluT4; Grillo et al., 2009; Reagan, 2005) and glucose metabolism enzymes (Grillo et al., 2009; Hoyer et al., 1993, 1996). Insulin is also involved in, for example, amyloid processing and packaging (Mullins et al., 2017), GABA receptor modulation (Trujeque-Ramos et al., 2018), potassium movement (Kleinridders et al., 2014), and tau processing (Gonçalves et al., 2019). Recently, there has been particular interest in the procognitive role of insulin as a cognitive modulator and, conversely, the cognitive impairment associated with impaired brain insulin signalling which has major clinical relevance.

Brain insulin has been shown to (i) be essential to spatial memory task performance (Ahmad & Al-Domi, 2018; McNay et al., 2010), (ii) improve declarative memory and increase connectivity between the hippocampus and prefrontal cortex (Benedict et al., 2004, 2011; Benedict & Grillo, 2018; Reger et al., 2008; Zhang et al., 2015), (iii) attenuate effects of hippocampal lesions (de Castro & Balagura, 1976), and (iv) modulate cerebral blood flow (Katakam et al., 2009; Wingrove et al., 2021). Moreover, GluT4 has also been shown to be an important regulator of hippocampal cognitive function (McNay & Pearson-Leary, 2020; Pearson-Leary et al., 2018; Pearson-Leary & McNay, 2016) including specific provision of glucose to neurons at times of increased activity (Ashrafi et al., 2017). GluT4 expression in the cerebellum has been reported as 0.3 pmol/mg (Vannucci et al., 1998), roughly 1–2 orders of magnitude lower than that of GluT1 and GluT3 (Vannucci et al., 1997); note also that as mentioned above, in contrast to GluTs 1 and 3, GluT4 expression varies widely across brain regions and is primarily not at the cell surface other than at times of acute cognitive/energetic demand (Ashrafi et al., 2017; Pearson-Leary et al., 2018). Dysregulation of brain insulin signalling is associated with cognitive impairment, including that associated with obesity (Greenwood & Winocur, 2001; Kaiyala et al., 2000; McNay et al., 2010; Ross et al., 2009; Winocur & Greenwood, 2005) where brain insulin levels may also be abnormal (Baskin et al., 1985; Gerozissis et al., 1993), and brain insulin resistance has been shown to cause brain energy metabolism impairment (Girault et al., 2019; McNay et al., 2000; Soares et al., 2019) associated with cognitive impairment and a number of neurodegenerative diseases (McEwen et al., 2002; Schulingkamp et al., 2000) – most prominently Alzheimer’s disease, where patients commonly show impaired brain insulin signalling even when not systemically insulin-resistant (Talbot et al., 2012). Increasing evidence has shown that insulin can be produced by the brain as well as being delivered from the pancreas via the bloodstream and blood–brain barrier (McNay & Recknagel, 2011). To understand the mechanisms by which insulin has procognitive effects within, e.g., the hippocampus, it is critical to know the level of insulin within the interstitial space of such regions, both at baseline and at times (such as during cognitive challenge) when insulin may be being actively released/modulated locally, as well as in conditions such as systemic insulin resistance. The latter continues to be somewhat controversial: while increased systemic insulin might perhaps be expected to be reflected in increased brain interstitial insulin (which would be consistent with central insulin resistance), some authors have suggested, based on measurements from the cerebrospinal fluid, that patients with systemic insulin resistance may in fact have reduced central insulin (Craft et al., 1998; Engel et al., 2022; Gray & Barrett, 2018).

Despite the importance of such measurements, only a handful of studies have monitored insulin concentration in the brain’s extracellular fluid in vivo. In the hypothalamus, microdialysis measurements showed rapid increases in insulin that were suggested to be from local synthesis and illustrated how fluctuations in ECF insulin concentration affect both metabolic and neurotrophic systems (Gerozissis et al., 1993, 1997, 1998; Orosco et al., 2000). Two studies have reported data on hippocampal ECF insulin in genetic mouse models of Alzheimer’s, but suffer from methodological issues (e.g., very short time between surgery and measurement; non-physiological perfusate composition) that make interpretation and use of the data challenging (Stanley et al., 2016; Wakabayashi et al., 2019); moreover, there are no data regarding basal or stimulus-altered interstitial brain insulin levels in control animals or humans. The field needs clear, physiologically obtained data on brain interstitial insulin levels across regions (most obviously, within the hippocampus) and across neural states (in particular, at baseline and during cognitive challenge): such data will permit understanding of the mechanisms by which insulin modulates metabolism and cognition and the ways in which these mechanisms are impaired in disease states, likely leading to novel therapeutic avenues. Metabolic regulation by insulin is expected to be particularly complex due to the compartmentalized nature of brain energy metabolism, and the interaction with other neuromodulation systems (Duarte, 2023) including that operated by adenosine (see Section 9.1).

## 5 | REDOX MOLECULES: TRANSCRIPTIONAL REGULATION, AGING, AND NEURODEGENERATION

### 5.1 | Intracellular compartmentation and transport of NAD^+^

Nicotinamide adenine dinucleotide (NAD^+^) and its reduced form NADH are an essential cofactor for enzymatic reactions involved in cellular energy metabolism. Furthermore, NAD^+^ serves as a substrate for enzymes that modulate protein acetylation, alter gene expression, participate in lipid synthesis, amino acid and sterol metabolism, inflammatory and stress responding, and are involved in aging processes. The intracellular distribution of NAD^+^ between the cytosol and subcellular organelles, particularly mitochondria, depends on both the type and metabolic state of the cell (Alano et al., 2007).

The levels of NAD^+^ in each discrete intracellular compartment are determined by two factors. First is the balance between the rate of NAD^+^ consumption and the rate of NAD^+^ generation. Second is the transport/exchange of NAD^+^ between the individual subcellular pools.

The major pathway that replenishes NAD^+^ in mammalian cells (NAD^+^ salvage pathway) is comprised from two enzymatic reactions. In the first step, the product of NAD^+^ degradation nicotinamide (Nam) is combined with phosphoribose pyrophosphate by nicotinamide phosphoribosyl transferase (Nampt) to generate nicotinamide mononucleotide (NMN). In the next step, NMN is converted to NAD^+^ in the presence of ATP by nicotinamide mononucleotide adenylyl transferase (NMNAT; Belenky et al., 2007). There are three isozymes of NMNAT (NMNAT1–3), each localized in a different cell compartment (NMNAT1 in the nucleus, NMNAT3 in the mitochondria, and NMNAT2 in the cytosol and Golgi; Jayaram et al., 2011). The presence of NMNAT2 in the Golgi apparatus suggests that this subcellular organelle has its own enzymatic machinery linked to NAD^+^ metabolism; however, it was shown that NMNAT also functions as a chaperone facilitating protein folding (Ali et al., 2012; Ocampo et al., 2013; Zhai et al., 2008).

The presence of NAD^+^ synthesizing enzyme in mitochondria led for many years to the assumption that mitochondrial and cytosolic NAD^+^ pools are relatively distinct from each other and NAD^+^ can be generated in the mitochondrial matrix (Ali et al., 2013; Pittelli et al., 2010; Yang & Shen, 2007). This notion was also supported by the fact that changes in mitochondrial reduction and oxidation state of pyridine nucleotides were detected without measurable changes of cytosolic NAD^+^/NADH ratio, thus suggesting compartmentalization of the two NAD^+^ pools (Chance & Williams, 1955; Pittelli et al., 2010; Sauve, 2008; Yang & Shen, 2007). However, in plants and yeast, a mitochondrial transporter specific for NAD^+^ was identified (Palmieri et al., 2009; Todisco et al., 2006).

In mammalian brain mitochondria, the movement of NAD^+^ across mitochondrial membranes was observed only under stress conditions when the mitochondrial permeability transition pore is activated (Bernardi, 1999; Kristian & Fiskum, 2004). This inner mitochondrial permeability pore is not specific for NAD^+^ and allows solutes of molecular weight up to 1500 Da to pass, thus leading to depolarization of the mitochondrial membrane potential (Bernardi, 1999). Although, this “mega” channel has a large conductance, the NAD^+^ release is relatively slow, taking several minutes to completely deplete the mitochondrial NAD^+^ (Kristian & Fiskum, 2004). However, the presence of an NAD^+^-specific channel in the inner mitochondrial membrane has several problems.

Although oxidized NAD is written in the form of a cation (NAD^+^), the molecule in the solution is negatively charged due to two negative phosphate groups. Thus, it has one negative net charge. Considering that the mitochondrial membrane potential is about −180 mV, the intramitochondrial NAD^+^ concentration would reach three orders of magnitude lower levels than the cytosolic one at the Nernst equilibrium. Since mitochondrial volume in the brain occupies only 3%–8% of the total cellular volume (Deutsch et al., 1979; Pysh & Khan, 1972), mitochondria should contain about 20 000 times less NAD^+^ than the cytosol, and thus about 0.005% of the total cellular NAD^+^. However, mitochondrial NAD^+^ levels are higher than cytosolic levels; the relative difference is cell-type-dependent (Sauve, 2008). In neurons, mitochondrial NAD^+^ pools represent about 50% of the total cellular NAD^+^, and in astrocytes only 25% (Alano et al., 2007). Mammalian mitochondria cannot synthetize NAD^+^ (Cambronne et al., 2016; Felici et al., 2013). Therefore, it is more likely that an NAD^+^-specific transporter is contributing to the replenishment of intramitochondrial NAD^+^ pools.

Indeed, recently three groups identified a soluble carrier SCL25A51/MACRT1 as the major mitochondrial NAD^+^ transporter in mammalian cells (Girardi et al., 2020; Kory et al., 2020; Luongo et al., 2020). SLC25A51 shows specificity to NAD^+^ when compared to other metabolites or the NAD^+^ precursor NMN (Girardi et al., 2020; Kory et al., 2020; Luongo et al., 2020). However, reports regarding NADH transport ability are conflicting. It is also not known whether SCL25A51 is a co-transporter or an exchanger and which metabolites are synergistically transported with NAD^+^. To ensure electroneutrality, and thus independence of the NAD^+^ transport from mitochondrial membrane potential, NAD^+^ should either be co-transported with a metabolite with positive charge or the transporter works as an antiporter, transporting a negative charged metabolite in the opposite direction, from the mitochondrial matrix into the cytosol. This is supported by data reported in studies with yeast and plant mitochondria, suggesting that the net charge of the NAD^+^ transport is negative since the NAD^+^ can be exchanged with ADP or AMP which carry several negative charges (Palmieri et al., 2009; Todisco et al., 2006). Replenishment of depleted mitochondrial NAD^+^ pools by supplying external NAD^+^ is slow, taking several minutes (Girardi et al., 2020; Kory et al., 2020; Luongo et al., 2020). Therefore, to determine the effects of the transporter on the dynamics of mitochondrial metabolism, the rate of NAD^+^ transport by SCL25A51 needs to be further studied and determined.

Additionally, it is not known whether SLC25A51 activity can be modulated by post-translational modifications, particularly acetylation. Mitochondrial deacetylase Sirtuin 3 (SIRT3) is NAD^+^-dependent and deacetylates several enzymes of the Krebs cycle, respiratory chain, and the ATP synthase (Waddell et al., 2021). Thus, NAD^+^ transport from the cytosol will have modulatory effects beyond influencing the rate of Krebs cycle metabolism but also on the rate of citrate generation, its transport into the cytosol, and downstream cytosolic acetyl-CoA generation by ATP-citrate lyase, affecting histone acetylation, gene expression, and lipid metabolism.

In conclusion, considering the essential role of NAD^+^ homeostasis for mitochondrial respiratory functions and downstream effects via NAD^+^-dependent SIRT3 deacetylation activity, further studies of the NAD^+^ exchange mechanisms between different subcellular compartments are crucial. These studies will have a significant impact on our knowledge related to cellular bioenergetic metabolism and may reveal novel regulatory pathways and therapeutic targets that might be related to mechanisms of neurotological diseases.

### 5.2 | What we do not know about NAD^+^ metabolism in the brain

Our understanding of how NAD^+^ changes in the human brain with age or disease remains rudimentary. Studies using magnetic resonance spectroscopy have determined that NAD^+^ levels decrease in the brain with normal human aging, probably due to the combined effects of a shift to its reduced form, NADH, and a decrease in the total (NAD^+^ + NADH) pool size (Bagga et al., 2020; Zhu et al., 2015). Moreover, a small study in Parkinson’s disease patients has provided preliminary evidence that supplementation with nicotinamide riboside may increase brain NAD^+^ concentration (Brakedal et al., 2022). These findings are consistent with studies on rodents (Yoshino et al. 2018). However, they remain to be replicated in larger studies, and their ultimate significance is far from clear as we do not know whether NAD^+^ metabolism is causally related to aging or neurodegenerative disease pathology, and if so, which cell types or compartments within a cell of interest are mediating the effects.

One of the most exciting findings to come out of preclinical studies on NAD^+^ precursors in rodents is an improvement in cognition across multiple models of Alzheimer’s disease (Gong et al., 2013; Green et al., 2008; Hou et al., 2018, 2021; Kim & Yang, 2017; Liu et al., 2013; Long et al., 2015; Rehman et al., 2021; Turunc Bayrakdar et al., 2014; Vakilinezhad et al., 2018; Wang et al., 2016; Xie et al., 2019; Yao et al., 2017). Only a few of these studies have measured NAD^+^ levels in the brain, and even in rodents, it remains unclear whether the NAD^+^ concentration in the brain is the relevant therapeutic target. Alternatively, systemic factors affected by NAD^+^ supplementation, such as changes in inflammatory cytokines or neuroinflammation (e.g., (Elhassan et al., 2019; Sims et al., 2018; Zhao et al., 2021; Zhou, Wang, et al., 2020)), endothelial function (Das et al., 2018; de Picciotto et al., 2016; Martens et al., 2018; Tarantini et al., 2019), or general improvements in endocrine function and nutrient handling (Yoshino et al. 2018) might produce neurological improvements as a secondary consequence. To date, the only published human trials on NAD^+^ supplementation in Alzheimer’s disease were small, used relatively low dose NADH (10 mg/day), and reached contradictory conclusions (Birkmayer, 1996; Rainer et al., 2000). However, a number of larger trials are announced or in progress and should shed light on the therapeutic potential of NAD^+^ supplementation in the coming years.

Changes in neurovascular coupling with NAD^+^ supplementation (Tarantini et al., 2019) are particularly intriguing as this process diminishes with normal aging and may relate to cognitive decline outside of any specific disease process (Tarantini et al., 2017). Supporting a more general effect of NAD^+^ boosting on brain health, oral, and intranasal precursors has also shown promise in models of ischemic injury, trauma, and ALS (Cheng et al., 2022; Klaidman et al., 2003; Park et al., 2016; Won et al., 2012; Ying et al., 2007; Zheng et al., 2022; Zhou, Zhu, et al., 2020). Notably, ALS has been linked to hyperactive forms of the NADase SARM1 (Gilley et al., 2021), and protective effects of nicotinamide riboside in combination with the polyphenol pterostilbene have been observed in a small trial with human ALS patients (Gilley et al., 2021), although the relative contributions of each molecule have not been resolved.

Protective effects of NAD^+^ supplementation have also been shown in models of DNA repair deficiencies with neurological effects, including ataxia telangiectasia (Fang et al., 2016), Werner syndrome (Fang et al., 2019), and Cockayne syndrome (Scheibye-Knudsen et al., 2014). Intriguingly, in the case of Cockayne syndrome in mice and in worms or mammalian cells modeling xeroderma pigmentosum group A, it has been shown that NAD^+^ deficiency arises at least in part due to hyperactivation of PARP1 secondary to DNA damage. Blocking PARP activity or restoring NAD^+^ by supplementation both alleviate symptoms in mice despite not addressing the underlying DNA repair defects (Fang et al., 2014; Scheibye-Knudsen et al., 2014). Whether this will hold true for Cockayne syndrome or other DNA repair disorders in human subjects remains to be seen.

One of the most intriguing observations surrounding the regulation of NAD^+^ metabolism in the brain is the connection between circulating levels of extracellular nicotinamide phosphoribosyltransferase (eNampt) and hypothalamic NAD^+^ concentration (Yoon et al., 2015). Nicotinamide phosphoribosyltransferase catalyzes the rate-limiting step in the NAD^+^ salvage pathway within cells but has been recognized for some time to also be secreted via a noncanonical pathway from immune cells and adipocytes. At least the portion secreted from adipocytes is now recognized to be contained within vesicles that can influence hypothalamic NAD^+^, ostensibly by transferring the enzyme into neurons (Yoshida et al., 2019). Moreover, daily injection of adipocyte-derived vesicles is sufficient to increase hypothalamic NAD^+^ and extend lifespan in mice (Yoshida et al., 2019). Whether the increase in hypothalamic NAD^+^ is the causal effect in longevity and whether a similar system operates in humans remain to be determined.

While all of these observations suggest that physiologically relevant changes in NAD^+^ concentration can have important consequences within the brain, there remain more questions than answers. Which cell types are experiencing deficits and/or responding to NAD^+^ precursors? And within a given cell, where does NAD^+^ matter? The identification of the mitochondrial NAD^+^ carrier (Girardi et al., 2020; Kory et al., 2020; Luongo et al., 2020) has opened up the possibility of probing specific changes in that compartment, but many more NAD^+^ pools exist (VanLinden et al., 2017) and likely have distinct functions. Finally, within a compartment, what are the critical NAD^+^-dependent processes that are being compromised or rescued? More than 80 years after the identification of NAD^+^ as a critical element of mammalian metabolism, we are still learning how much we have left to learn.

## 6 | FATTY ACID TURNOVER DURING DEVELOPMENT, AGING, AND DISEASE

### 6.1 | Fatty acid synthesis and oxidation after developmental injury: Can we find a balance?

Developmental brain injury is a major contributor to long-term neurodevelopmental delays (Chau et al., 2013; Schiller et al. 2018). Advanced structural and biochemical imaging associates these neurodevelopmental delays with long-lasting maturation failure and a disrupted trajectory of growth. At the cellular level, neural progenitor cells respond to neonatal brain injury by proliferation, migration, and delayed differentiation (Salmaso et al., 2014; Zonouzi et al., 2015). This recovery phase is metabolically expensive, imposing additional energy demands and disrupting highly orchestrated brain development and maturation (Folmes & Terzic, 2016). Therefore, there is a critical need to delineate acute post-injury and sustained long-term metabolic adaptations after developmental brain injury. An improved understanding of the metabolic plasticity following injury could lead to improved neurologic outcomes.

The brain is the second-most lipid-rich organ with most lipids synthesized in the brain *de novo*. Fatty acids (FAs), the underpinning of lipids, are essential in the developing brain for myelination, neurogenesis, and lipid membrane turnover (Clandinin, 1999; Poitelon et al., 2020). Endogenous *de novo* FA synthesis in the brain pre-dominates in the first years of life, concurrent with rapid structural brain growth (McKenna et al., 2015; Steiner, 2019). These FAs are essential building blocks of diverse brain lipids such as sphingolipids, ceramides, and glycolipids, to name a few. Endogenous FA synthesis in the brain is especially high during the last trimester of gestation and early life, as FAs are crucial for neurogenesis, synaptic formation, myelination, and lipid membrane formation and turnover (Chirala et al., 2003; Edmond et al., 1998; McKenna et al., 2015; Volpe et al., 1973). Synthesis of FAs is carried out by FA synthetase (E.C.2.3.1.85), which is expressed in all brain cells (neural precursor and stem cells, astrocytes, oligodendrocytes, microglia, and neurons). FA synthetase catalyzes the entire synthesis of FAs using the substrates acetyl-CoA and its carboxylated product malonyl CoA. A major product is C16 palmitic acid, which can be further elongated and desaturated (addition of double bonds). Studies in adult rodents have shown that either pharmacologic or genetic inhibition of FA synthesis leads to significant i) decrease in the proliferation of neural progenitor cells; ii) decrease in neurogenesis at the neurogenic niches; and iii) alteration in myelin lipid composition and stability (Knobloch, 2017; Knobloch et al., 2013). Whereas global (whole body) fatty acid synthase-null mutants die *in utero*, and mutations or heterozygote removal of fatty acid synthase results in significant intellectual disabilities (Bowers et al., 2020; Chirala et al., 2003). By using human embryonic stem cell-derived fore-brain organoids, it was demonstrated that loss of fatty acid synthase disrupts radial glia polarity and results in microcephaly (Gonzalez-Bohorquez et al., 2022). Despite this emerging evidence, the effect of cell-specific deletion, mutation, or altered expression of fatty acid synthase during brain development or recovery following developmental injury is not known.

Although a majority of saturated and monosaturated FAs found in brain lipids are produced by *de novo* synthesis (Edmond et al., 1998), the brain does rely on import of some of the essential FAs. Long-chain polyunsaturated fatty acids omega-3 and omega-6 are essential components of signaling and plasma membrane formation; hence, the brain relies on import of these polyunsaturated fatty acids from systemic circulation (Cunnane et al., 2000; Ebert et al., 2003). Recent evidence demonstrates that in addition to systemic supply of these polyunsaturated fatty acids, brain Acsl6 is essential for retention of docosahexanoic acid in the brain (Fernandez et al., 2021).

In contrast to the adult brain, which uses 75% of its primary fuel (glucose) for synaptic transmission, the developing brain is more energy-expensive, relying on a constant supply of fuel to meet high-energy demand and a high rate of macromolecular synthesis required for growth and maturation. Specifically, neurogenesis, synaptogenesis, and myelination which occur during early brain development require extensive *de novo* synthesis of cholesterol, fatty acid esters, sphingolipids, and phospholipids. These synthetic processes exceed glucose availability (Carey, 1975; McKenna et al., 2015). Therefore, the necessary carbon skeletons can be furnished by fatty acid uptake from systemic circulation. Uptake of long-chain saturated and unsaturated fatty acids across the blood–brain barrier has been reported decades ago (Dhopeshwarkar et al., 1972; Dhopeshwarkar & Mead, 1969), but it remains a subject of ongoing debate and depends on age (immature, prior to or during myelination vs adult) and fatty acid chain length; i.e., the brain is capable of synthesizing *de novo* non-essential fatty acids but depends on transport of essential fatty acids (omega-3 and omega-6; Dhopheswarkar & Mead, 1973; Edmond et al., 1998; Miller et al., 1987; Rapoport et al., 2001; Smith & Nagura, 2001; Spector, 1988). While brain fatty acid uptake occurs within minutes (Kuge et al., 1995; Miller et al., 1987; Smith & Nagura, 2001; Spector, 1975; Sun & Horrocks, 1971), two mechanisms for fatty acid transport are proposed: 1, diffusion; 2) transport of fatty acids via specific protein-mediated transporters, and several protein families. Fatty acid transport proteins, fatty acid binding proteins, and fatty acid translocase/CD36 have been identified to play a role in facilitating fatty acid transport in the brain (see extensive reviews (Liu et al., 2010; Mitchell & Hatch, 2011)). Essential and non-essential fatty acids taken up into the brain via the blood–brain barrier and de novo synthesized brain non-essential fatty acids are subject to further metabolism, primarily incorporation into complex lipids (Dhopeshwarkar & Subramanian, 1975; Miller et al., 1987).

In the adult brain, fatty acids undergo mitochondrial β-oxidation to generate acetyl-CoA, which becomes a building block for *de novo* brain lipid synthesis or enters the Krebs cycle to generate Krebs cycle intermediates, amino acids, and ATP. The presence of fatty acid oxidation in the brain was reported decades ago, and it was determined that under normal physiological conditions, brain fatty acid oxidation (determined by ^14^CO_2_ quantification) is low (Carey, 1975; Miller et al., 1987; Uda et al., 1981; Vignais et al., 1958). However, using ^14^C labeled fatty acids, these studies reported that label incorporation was detected in glutamate, glutamine, GABA, and amino acids (Miller et al., 1987; Uda et al., 1981). These studies were often performed using whole brain homogenates without accounting for age, brain region, and cell-specificity. By using the same technique in primary cell cultures, it was shown that only intact astrocytes were able to use medium-chain fatty acids (octanoate) and long-chain fatty acids (palmitate) for CO_2_ production (Edmond et al., 1987). Recent studies attempted to quantify metabolism of medium-chain fatty acids using ^13^C-labeled octanoate via NMR ex vivo in adult rat brain (Ebert et al., 2003) and with gas-chromatography-mass spectrometry in adult mouse cerebral cortex slices (Andersen et al., 2023). These studies demonstrated that labelling of glutamine was significantly higher than that of glutamate, indicating that octanoate oxidation is present in astrocytes, further corroborating earlier work (Edmond et al., 1987). However, there continues to be a lack of data regarding the extent of complete vs incomplete fatty acid oxidation or the amount of ATP generated by fatty acid oxidation.

With advances in molecular biology, there is a renewed interest in fatty acid oxidation with the focus on cell-specificity and pathological conditions. Recent studies demonstrate that carnitine palmitoyltransferases which mediate entry of long-chain acyl-CoAs into mitochondria for subsequent β-oxidation are present in astrocytes and are essential for quiescent neural progenitor and glioblastoma cells (Jernberg et al., 2017; Kant et al., 2020; Knobloch, 2017; Knobloch et al., 2017; Stoll et al., 2015). Interestingly, the neonatal brain contains a high density of immature cells (neural progenitor cells), and this population has been shown to increase after injury (Buono et al., 2015). Whether fatty acid oxidation is altered following neonatal brain injury remains to be determined. In contrast, several recent studies show that acylcarnitines, which are generated via carnitine palmitoyltransferases and reflect fatty acid mobilization for subsequent oxidation, are increased in aging and following stroke (Loppi et al., 2023; Mi et al., 2023).

The highly orchestrated process of fatty acid oxidation and de novo fatty acid synthesis in the brain with regional, temporal, and cell-specific characteristics following the injury remain elusive to date. Following injury during development, this fine-tuned machinery characterized by extensive fatty acid synthesis and fatty acid oxidation restricted to neural progenitor cells is disrupted; hence, one can hypothesize that injury may results in increased FA oxidation during the acute period. What remains unknown is the extent to which FA β-oxidation after developmental brain injury is redirected towards energy production at the expense of FA and lipid synthesis, thus leading to structural dysmaturation. Furthermore, cell-specific discoordination of FA synthesis and FA β-oxidation after neonatal brain injury has not been delineated. Understanding of timing, duration, and cell specificity of this process may help scientists and clinicians test whether increasing supply of FAs or their precursors (for example acetate or docosahexanoic acid) in acute or sub-acute stage may help brain recovery, as well as developing therapies that enhance metabolic plasticity by affecting enzymatic machinery. An improved understanding of the imbalance between FA synthesis and β-oxidation of long-chain FAs after developmental brain injury could lead to the development of targeted interventions to restore coordination of these metabolic pathways. The end result could be improved brain bioenergetics and neurologic outcomes, without the debilitating side effects commonly seen in manipulations of glucose metabolism.

### 6.2 | Emerging roles of lipid droplets in astrocytes: Neuroprotection against disease progression?

Although almost 60% of the dry weight of the brain is lipids (O’Brien & Sampson, 1965), the production of energy in the brain depends mainly on the supply of glucose from the bloodstream. Energy-rich fatty acids (FAs) account for, under maximal conditions, only ca. 20% of the total energy expenditure in the adult brain; this takes place primarily in astrocytes (Panov et al., 2014). Despite not being the main energy substrates, lipids are still crucial for normal brain function. They are building blocks of the brain cell membranes and myelin, enabling efficient, rapid conduction of signals, and precursors of signaling molecules (Barber & Raben, 2019). Dysregulation of brain lipid homeostasis has been linked to neuropathology, but the functional importance of lipid droplets in the brain has emerged only recently (Bailey et al., 2015; Liu et al., 2015). Lipid droplets are cytoplasmic lipid storage organelles, the centre of lipid homeostasis, and play important roles in intracellular lipid transport, lipid turnover, and stress response (Olzmann & Carvalho, 2019). They are not often detected in the adult, healthy brain, but accumulate in the brain, mainly in neuroglia (astrocytes, microglia, ependymal cells, and oligodendrocytes), during development, aging, neurological disorders, including neurodegeneration, neuroinflammation, stroke, trauma, and cancer (Farmer et al., 2020; Marschallinger et al., 2020; Ralhan et al., 2021; Smolič, Zorec, & Vardjan, 2021; Yang et al., 2022). Lipid droplets have been linked to neuroprotection but might also contribute to disease progression (Farmer et al., 2020; Ralhan et al., 2021; Smolič, Zorec, & Vardjan, 2021; Yang et al., 2022). Inhibition of glial lipid droplet formation can delay neurodegeneration (Liu et al., 2015). Thus, lipid droplets in the brain might represent a novel drug target and disease marker in the future because they are already present before or at the onset of neurodegeneration (Liu et al., 2015). The molecular mechanisms that trigger the change from their neuroprotective role to toxicity are far from clear, highlighting the importance of future research concerning the biology of lipid droplets and their composition and regulation in different brain cells in health and disease.

Astrocytes, key brain cells maintaining metabolic homeostasis in the brain, respond to different stressors, typical for brain pathologies, with lipid droplet accumulation (Smolič, Tavčar, et al., 2021; Smolič, Zorec, & Vardjan, 2021). In resting astrocytes, the turnover of lipid droplets seems to be rapid (lipid droplets are only rarely detected) but still essential for the maintenance of the cell cycle and/or survival. Lipid droplet turnover in astrocytes is affected by metabolic and hypoxic stress, causing lipid droplet accumulation (Smolič, Tavčar, et al., 2021; Smolič, Zorec, & Vardjan, 2021). Under starvation, autophagic breakdown of the cell membranes might supply lipid droplets with membrane FAs, leading to lipid droplet accumulation and a shift from glucose metabolism to lipid droplet-derived FA oxidation (Rambold et al., 2015), enhancing cell survival under starvation. Also, hypoxia and excess exogenous FAs and L-lactate, both of which accumulate in the brain during hypoxic stress, trigger lipid droplet accumulation in astrocytes. Lipid droplets probably buffer build-up of FAs during hypoxia (hypoxia activates FA synthesis in astrocytes (Lee et al., 2015)), which if not temporarily stored in lipid droplets, can generate a lipotoxic environment (Bailey et al., 2015; Islam et al., 2019). Cytoplasmic amyotrophic lateral sclerosis-linked protein TDP-43 inclusions in astrocytes triggers accumulation of lipid droplets, suggesting a stress response, linking astroglial lipid droplets to TDP-43 proteinopathies (Velebit et al., 2020). Lipid droplet turnover in astrocytes is also under the control of noradrenergic inputs (Smolič, Tavčar, et al., 2021). Astrocytes respond to long-term exposure to the stress response neuromodulator, noradrenalin, by accumulating lipid droplets (Smolič, Tavčar, et al., 2021). The molecular mechanisms underlying changes in lipid droplet turnover in astrocytes in stressed brain and how they affect the activity of astrocytes and astrocyte–neuron networks are not yet known and need further attention.

Recently, new understanding of astrocyte–neuron metabolic coupling has emerged, describing the transfer of lipids from neurons to astrocytes leading to lipid droplet accumulation in astrocytes (Ioannou et al., 2019; Liu et al., 2017). It has been suggested that the transport of L-lactate from astrocytes to neurons via an astrocyte–neuron lactate shuttle triggers in stressed, overstimulated neurons de novo synthesis of FAs from L-lactate. L-Lactate is decarboxylated in neuronal mitochondria, and the resulting acetyl-CoA generates FAs. Because FA overload in neurons is associated with the lipid peroxidation chain reaction and generation of reactive oxygen species, which may lead to lipotoxicity (Bailey et al., 2015), FAs are transferred from neurons to astrocytes in apolipoprotein E and D-like particles, which are then endocytosed by astrocytes (Ioannou et al., 2019; Liu et al., 2017). In astrocytes, neuronal FAs are temporarily stored in lipid droplets to protect neurons (and astrocytes) from lipotoxicity (Ioannou et al., 2019; Liu et al., 2017). Neural activity triggers oxidation of lipid droplet-derived FAs in astrocytes (Ioannou et al., 2019) because, unlike neurons, astrocytes have the capacity to fight overproduction of ROS during β-oxidation (Schönfeld & Reiser, 2013). These recent data on astrocyte–neuron metabolic coupling leave many open questions, such as why would hyperactive neurons produce their own FAs from astroglial L-lactate and transport them to astrocytes for energy production when glucose levels are not limiting? The transport seems to be crucial, given that the inability of stressed neurons to transport lipids for lipid droplet formation in astrocytes leads to neurodegeneration (Liu et al., 2015; Moulton et al., 2021).

## 7 | METABOLIC DYSFUNCTION IN AGING

### 7.1 | Metabolic tuning throughout the anatomy of aging neurons

Neurons are highly polarized cells with thin, long structures (dendrites and axons) that have even thinner evaginations (dendritic spines and synaptic terminals, respectively) constituting an anatomical scaffold for neurotransmission. Due to physical constraints and limited metabolite diffusion, such anatomic regions must rely on local and highly efficient energy production to cope with moment-to-moment energy demands. These requirements likely result in compartment-specific metabolic adaptations. In synaptic terminals, for example, glycolytic enzymes can undergo phase separation and aggregate upon strong stimulation (Jang et al., 2016, 2021). In presynaptic compartments, glucose transporters (GLUT4) can be translocated to the plasma membrane in response to highly localized energy demand rather than the canonical insulin signaling observed in peripheral tissues (Ashrafi et al., 2017).

The concerted actions of neuronal glycolysis and oxidative phosphorylation can support rapid axonal firing and neurotransmission (Yellen, 2018). Nevertheless, the metabolic autonomy of stimulated adult neurons and their synapses remains debated; i.e., whether their mitochondria are predominantly fuelled by endogenously produced pyruvate or astrocyte-derived lactate (Alberini et al., 2018; Díaz-García & Yellen, 2019; Dienel, 2019; Magistretti & Allaman, 2018). Neuronal glycolysis appears to be particularly coupled to the restoration of ionic gradients across the plasma membrane (Díaz-García et al., 2021; Lujan et al., 2016; Meyer et al., 2022). Its activation upon synaptic stimulation can overwhelm the capacity of mitochondria to oxidize the resulting pyruvate, which is manifest as transient increases in cytosolic NADH and lactate within the cell (Díaz-García et al., 2017). However, the balance between glycolysis and oxidative phosphorylation may not be identical in all neuronal compartments and may be influenced by the specific energy demands in excitatory principal neurons and inhibitory interneurons resulting from cell signaling, ion pumping, and other events related to neurotransmission (Hall et al., 2012; Kann et al., 2014; Pulido & Ryan, 2021).

The need for glycolytic ATP may decline with age because of increased reliance on mitochondrial metabolism as neurons and their synapses mature (Cruz et al., 2022; Goyal et al., 2014; Lujan et al., 2021). Nevertheless, this metabolic switch may not occur to the same degree in all types of neurons and subcellular compartments, especially in those that may lack mitochondria at any given time, e.g., dendritic spines and synaptic terminals (Chavan et al., 2015; Kasthuri et al., 2015). It is also known that energy metabolism operates in coordination with enzymes like adenylate kinase and creatine kinase (Erecinska et al., 2004). Although there is increasing evidence of its relevance for synaptic integrity and activity (Chen et al., 2021; Justs et al., 2022), the interplay between the ATP buffering/regenerating systems and glycolysis/oxidative phosphorylation is not fully understood in the context of neuronal activity and neurotransmission.

The fuel preference and the activity-dependent regulation of energy metabolism throughout the anatomy of aging neurons remains an enigma. As the brain ages, impaired glucose utilization and hypoglycemic episodes are evident, both of which increase the risk of cognitive decline (Abdelhafiz et al., 2015; Mosconi et al., 2008; Neth & Craft, 2017). Learning and memory, in particular, entail additional energy needs due to the protein synthesis necessary for the enlargement of synaptic contacts (Li & Van Rossum, 2020), which is largely supported by mitochondria (Rajgor et al., 2021; Rangaraju et al., 2019) and likely requires the oxidation of more than one metabolic fuel (Descalzi et al., 2019).

Metabolic dysfunction is one of the hallmarks of aging (Mattson & Arumugam, 2018) and, critically, dendrites and axons are prone to deteriorate during this process (Adalbert & Coleman, 2013; Bloss et al., 2011), despite little-to-no loss of neuronal somata (Bishop et al., 2010; Castelli et al., 2019). Therefore, more quantitative studies with sufficient spatiotemporal resolution, performed at near-physiologic conditions or in vivo, are required to reveal differences in the metabolic state at rest and during stimulation between the soma and other anatomic regions in the neuron. We look forward to learning how the balance between neuronal glycolysis and oxidative phosphorylation in dendrites and axons/synaptic terminals is regulated in response to various regimens of neuronal activity during aging, and whether there might be compensatory mechanisms through the expression of ATP buffering/regenerating systems, the use of alternative fuels, and/or metabolic cooperation with other neural cells.

### 7.2 | Mitigating cognitive decline through astroglial metabolism: Towards the noradrenergic hypothesis of neurodegeneration

The global population, particularly in the West, is aging rapidly, and cognitive decline and dementia are becoming major challenges. Clinical determination of dementia relies on progressive deterioration of two or more cognitive functions, including, but not limited to, language, memory, personality, executive function, and behaviour, sufficient to cause occupational or social impairments (Tarawneh & Holtzman, 2012). Alzheimer disease (AD), characterized by brain atrophy, extracellular deposits of amyloid plaques, and intracellular cytoskeletal neurofibrillary tangles, accounts for up to 80% of all dementia diagnoses.

On January 6, 2023, The US Federal Drug Administration conditionally approved and on June 6th 2023 fully approved a new drug (lecanemab, Leqembi^™^) to treat AD that very moderately slows cognitive decline in people with early-stage AD (van Dyck et al., 2022). It was developed by a Japanese company (Eisai), which produced the first symptomatic treatment for AD 25 years ago. Why did it take such a long time? The mechanism of action of lecanemab, a monoclonal antibody, is similar to that of aducanumab, previously conditionally approved for AD (Aduhelm, Biogen, and Eisai), by acting on and reducing β-amyloid deposits (Sevigny et al., 2016; van Dyck et al., 2022). However, clinical trials of both monoclonal antibodies revealed significant adverse events (brain swelling or brain bleeding); hence, treatment for AD remains an important unmet medical need, affecting millions of people worldwide.

The mechanisms leading to AD are unclear, and the pathophysiologic processes may start decades before symptoms appear (Braak & Del Tredici, 2011; Rodriguez-Vieitez et al., 2016). Several hypotheses underlying the pathogenesis of AD have been considered, including the amyloid cascade, the neurovascular, tau propagation, and the mitochondrial and the cholinergic hypotheses. The pathogenesis in all of these may involve various levels of neuroinflammation (Heneka et al., 2015). The cholinergic hypothesis is the oldest theory and posits the demise of cholinergic neurons in the nucleus basalis of Meynert (Slater & Wang, 2021), reported almost 50 years ago (Davies & Maloney, 1976), leading to a corresponding cholinergic failure and impairment of memory, attention, and learning (Lemière et al., 1999). Why cholinergic neurons degenerate is unclear, but this is associated with diminished levels of nerve growth factor, which maintains neurogenesis (Giacobini et al., 2022). The reduced levels of nerve growth factor are possibly due to altered astrocyte regulation of plasmin, an enzyme involved in the processing of nerve growth factor (Giacobini et al., 2022). This function is consistent with astrocytes being key to providing homeostasis in the central nervous system (CNS; Verkhratsky & Nedergaard, 2018) and being a target for new drug development (Verkhratsky et al., 2023).

Astrocytes are also involved in AD through neurodegeneration of locus coeruleus neurons (Leanza et al., 2018), the prime source of noradrenaline in the CNS, and play a fundamental role in many functions, including attention, arousal, sleep/wakefulness, and consciousness, as well as learning and memory (Benarroch, 2009). Locus coeruleus demise contributes to the loss of neural reserve in neurodegenerative disorders (Wilson et al., 2013). Why locus coeruleus neurons degenerate is unknown. Their localization near the 4th ventricle exposes them to a harmful environment, and they are uniquely susceptible to oxidative stress, possibly due to their relatively high energetic needs (Sanchez-Padilla et al., 2014).

Awareness of the development of deficits in the noradrenergic system decades before detecting symptoms of AD (Braak & Del Tredici, 2011) prompted development of strategies to increase noradrenaline levels in the CNS (Slater & Wang, 2021), either by attenuating its degradation via inhibitors of monoamine oxidase or inhibiting the uptake from the synaptic/extracellular space via membrane transporters. However, all these attempts failed to be clinically effective (Feinstein et al., 2016; Slater & Wang, 2021). Locus coeruleus—noradrenaline dysfunction in AD likely involves impairments of mechanisms that are downstream of different adrenergic receptors on various neural cell types, including astrocytes, which express practically all types of adrenergic receptors (Hertz et al., 2010). Activation of adrenergic receptors mediates many functions, including regulation of metabolism and in particular aerobic glycolysis (Prebil et al., 2011), with the end product being lactate. This process was discovered in cancer cells by Warburg, who considered that mitochondrial metabolism is altered in cancer cells (Warburg, 1956). However, astrocytes do exhibit mitochondrial metabolism linked to the production of cholesterol (Mauch et al., 2001) and lipid synthesis, which is regulated by noradrenaline (Smolič, Tavčar, et al., 2021).

In summary, although the cholinergic and the noradrenergic systems appear impaired in AD and both involve astrocytes as an intermediary cell type, the extent of noradrenergic cell loss in the locus coeruleus likely exceeds that of cholinergic neurons in AD (Zarow et al., 2003), consistent with a recent report showing that acetylcholine and noradrenaline differentially regulate hippocampus-dependent spatial learning and memory (de Leo et al., 2023), supporting the noradrenergic hypothesis of AD. Therefore, mitigating the mechanisms mediated by noradrenaline in AD may represent a new target to generate better drugs to prevent or eventually treat AD.

## 8 | BRAIN DISORDERS, INJURY, INFLAMMATION – METABOLISM-BASED THERAPEUTICS

### 8.1 | FDG-PET interpretations and cell type-specific utilization of glucose and auxiliary fuels in health and disease

Several glucose transporters have been cloned and characterized, and it is currently accepted that GLUT1–4 are the functional proteins transporting glucose within the brain. While the endothelial glucose transporter 1 is necessary for glucose transport via the blood–brain barrier, little is known about the role and function of Glut1 in astrocytes. Glut1 found in microvessels has a much higher molecular weight, namely, 55 kD, compared to the smaller 45 kD astrocytic Glut1, which is due to differences in glycosylation. While deletion of Glut1 is lethal in mice, it was recently reported that deletion of Glut1 in GFAP-promoter active cells paradoxically improved glucose metabolism (Ardanaz et al. 2022) and no signs of behavioural epileptic seizures were seen (Maite Solas, personal communication). In the Glut1 delta GFAP mice, brain FDG-PET signals were unexpectedly higher than in wild-type mice without any changes in CSF glucose or cognitive alterations. These puzzling high FDG-PET signals reveal our limits of information gained from this standard and clinically highly useful technique, namely, our inability to discern the contribution of different cell types to the signal and to distinguish between effects of alterations in glucose transport vs. hexokinase activity to FDG-PET signals, indicating glucose utilization. In addition, FDG-PET does not consider the effects of glycogen breakdown on glucose metabolism. Glycogenolysis is well-known to occur during stress, which is expected to be high during the imaging procedure. This influence of glycogen turnover on procedures measuring glucose utilization using labelled 2-DG or FDG was already mentioned by Sokoloff (Sokoloff et al., 1977) and was highlighted in 2023 (Dienel et al., 2023). For example, epileptogenic tissue that appears to be structurally normal by MRI often shows high-frequency oscillations in stereo-EEG measurements and would be expected to show high glucose utilization, but FDG-PET signals are unexpectedly low (O’Brien et al., 1997). Increased glycogen breakdown in astrocytes during stressful imaging procedures is likely to reduce glucose utilization, potentially specifically in epileptogenic tissue where glycogen levels are high and thus provides an explanation for apparent low brain glucose utilization (Dienel et al., 2023). On the other hand, if Glut1 delta GFAP mice breakdown less glycogen during the PET procedure than their wild-type counterparts, blood glucose utilization would be increased, although overall hexose consumption by the brain may be the same. A theoretical meta-analysis also indicated that during brain activation, blood glucose does not provide sufficient fuel for neurotransmitter cycling (glutamate/GABA-glutamine cycling) due to limited glucose transport via the blood–brain barrier. Rather, astrocytes may use glycogen as fuel to spare of glucose for neuronal consumption (Rothman 2022). New spectrophotometric methods to assess glycogen metabolism are urgently needed, especially as many neurological disorders show aberrations in glycogen levels.

The deficit in knowledge about the role of astrocytic Glut1 is most obviously a caveat for the treatment of Glut1 deficiency syndrome, a rare disorder. The syndrome is often diagnosed due to the occurrence of seizures in toddlers, but there are many other neurological and peripheral symptoms in this genetic condition, with one of the most debilitating being paroxysmal motor movements (Klepper et al., 2020). For children with Glut1 DS, the first line treatment is ketogenic diet. This dietary regime induces the formation of ketone bodies in the liver, which largely serve as auxiliary fuels in neurons, but also have many other beneficial metabolic, anti-oxidant, and anti-inflammatory functions. Ketogenic diets can be supplemented with medium-chain triglycerides which are highly ketogenic but also provide medium-chain fatty acids that are preferentially metabolized, in experiments using rodents, by astrocytes (Andersen et al., 2023). Thus, a ketogenic diet with purely long-chain fats is expected to mostly address the apparent deficit of glucose transport via the BBB and help provide fuel for neurons. However, it is unknown to which extent fuel is lacking in astrocytes, which if affected is expected to benefit more from medium-chain fatty acids than ketone supplementation. Similar questions about the impacted cell types and optimal treatments arise in other neurological disorders where glucose hypometabolism is apparent, such as Alzheimer’s and Parkinson’s disease, depression, and epilepsy.

In summary, there is a high need for better research methods and more detailed research to understand the detailed biochemical underpinnings of cell-specific impairments in energy metabolism in vivo. More knowledge is expected to enable personalized medicine and specific targeting of certain brain cell types with auxiliary fuels for optimal treatment.

### 8.2 | What are the mechanisms that link brain glycogen metabolism and pathology?

Glycogen is a branched polymer of glucose that constitutes the only carbohydrate reservoir in mammals. Its concentration in the brain is low compared to that of other tissues like the liver or the skeletal muscle, but in the recent years, the contribution of glycogen metabolism to normal brain functioning has become evident (Dienel, 2019). Indeed, this polysaccharide plays important roles in synaptic plasticity, learning, and memory (Alberini et al., 2018; Duran et al., 2013; Duran, Gruart, López-Ramos, et al., 2019; Suzuki et al., 2011). Although it was widely believed that brain glycogen is stored exclusively in astrocytes, it is now clear that neurons also have an active glycogen metabolism that contributes to brain function (Byman et al., 2021; Duran, Gruart, Varea, et al., 2019; Saez et al., 2014).

Furthermore, alterations in brain glycogen metabolism are associated to several neurologic conditions, including epilepsy, certain neurodegenerative diseases, or ischemia-induced damage. Glycogen is increased in the brain of epileptic patients (Dalsgaard et al., 2007) as well as after treatment with epileptogenic drugs like methionine sulfoximine (Seo et al., 2022; López-Ramos et al., 2015). Epileptic seizures result from an imbalance between glutamatergic (excitatory) and GABAergic (inhibitory) transmission. Disturbances in extracellular potassium are an important causal factor in epilepsy, and astrocytic glycogen fuels potassium reuptake by astrocytes (Xu et al., 2013). Thus, it has been hypothesized that impaired glycogenolysis in astrocytes compromises potassium reuptake and increases susceptibility to epilepsy (DiNuzzo et al., 2015). Mice devoid of brain glycogen have an increased susceptibility to epilepsy, which seemed to corroborate this hypothesis (López-Ramos et al., 2015). However, mice devoid of glycogen specifically in astrocytes do not present an epileptic phenotype (Duran et al., 2020). Thus, the connection between the lack of glycogen and epilepsy could be due to neuronal glycogen, in particular in GABAergic interneurons. Strikingly, Lafora disease, a rare neurodegenerative condition characterized by the accumulation of glycogen in astrocytes and neurons, also courses with epilepsy (Duran et al., 2014). Interestingly, neuronal glycogen accumulation underlies the epileptic phenotype of the disease (Duran et al., 2021). Another characteristic of Lafora disease is the presence of a severe neuroinflammation. In this case, this pathologic trait is driven mainly by the accumulation of glycogen in astrocytes (Duran et al., 2021). Again, the mechanisms that drive neuroinflammation upon astrocytic glycogen accumulation are not clear yet. Similarly, impaired glycogenolysis after reperfusion in ischemic stroke results in glycogen accumulation, which contributes to brain damage (Cai et al., 2020). Again, it has been hypothesized that the lack of astrocytic glycogen-derived glucose aggravates oxidative damage after reperfusion (Guo et al., 2022). However, in the light of the toxic effects of excessive glycogen identified in Lafora disease, the accumulation of glycogen itself could be harmful in these conditions.

In summary, the mechanisms by which impaired glycogenolysis and/or the accumulation of excessive glycogen in neurons and astrocytes induce pathology are not properly known yet. Elucidation of these molecular mechanisms would yield helpful information in the search of treatments for certain types of epilepsy, ischemia-induced damage, and for Lafora disease, as well as other more common neurodegenerative conditions that course with the accumulation of glycogen in neural tissues (Duran, Gruart, López-Ramos, et al., 2019).

### 8.3 | Static vs. dynamic impairment in energy metabolism following acute brain injury

Steep gradients in O_2_ and substrate availability across an ischemic penumbra are prominent features in stroke as well as other acute brain injuries with pockets of microvascular compression from mass occupying lesions, hematomas, and oedema (Abate et al., 2008; Astrup et al., 1981; Chen et al., 2011; Coles et al., 2004; Paschen et al., 1992). Detailed methods to map the ischemic penumbra have been developed, and preclinical and clinical studies have examined large numbers of molecular pathways modified by reduced oxygen and glucose availability. However, there is a well-documented failure in development of effective stroke therapeutics that are based on this large body of literature (Landis et al., 2012; Macrae & Allan, 2018; McCabe et al., 2018). There has been a growing realization that gradients of metabolic depletion in the injured brain are not static. Rather, there are dynamic events that massively distort energetic demand and supply, and it is these superimposed events, rather than background metabolic status by itself, that determine the fate of neurons to live or die.

Spreading depolarization is a wave of pathologic electrical activity that propagates through vulnerable tissue in the injured brain (Dreier, 2011; Hartings et al., 2017; Leao, 1944). SD propagates via feed-forward activation of voltage-gated and ligand-gated ion channels, resulting in massive net displacement of ions (Somjen, 2001). Defending and restoring ion gradients is energy-dependent, requiring activity of plasmalemmal Na/K-ATPase among other ATP-consuming ion pumps and cellular processes (Mies & Paschen, 1984; Selman et al., 2004; Unekawa et al., 2018). The energetic challenge can be compounded by paradoxical vascular responses to SD, depriving tissue of blood flow when it needs energy most (Dreier, 2011; Dreier et al., 2013; Hinzman et al., 2014; Menyhárt et al., 2018). Crucially, spreading depolarizations continue to occur intermittently over the hours and days following stroke. A number of pivotal studies have identified spreading depolarization as a key mediator contributing to secondary injury progression and worse clinical outcomes (Back et al., 1996; Dohmen et al., 2008; Dreier et al., 2017, 2022; Foreman et al., 2022; Hartings et al., 2011; Hartings, Bullock, et al., 2011). Many groups are beginning to pursue the question of whether blocking spreading depolarization is feasible, but there are challenges to this approach (Helbok et al., 2020; Shuttleworth et al., 2020). An urgent gap in the field is whether metabolic consequences of spreading depolarization in injured brain could be understood and exploited to develop complementary treatment strategies. *A mechanistic understanding of ATP generation and consumption could be leveraged to devise and test interventions to augment supply and/or limit demand in the critical periods when spreading depressions occur*.

To address this gap, new tools are needed, including robust, real-time reporters of cellular ATP levels and metabolite utilization that can be compared under different levels of spreading depolarization-induced energetic stress. In addition, there is an opportunity to adapt existing technologies. For example, increasing the sampling rate of microdialysis studies could allow the detection of metabolic consequences of spreading depolarizations, as these brief intermittent episodes are currently greatly underestimated with standard integration times (Pinczolits et al., 2017; Rogers et al., 2017). Similar considerations could apply to metabolic imaging methods that traditionally sample single time points, which could be modified to capture dynamic spreading depolarization events (Dienel et al., 2001). Finally, spreading depolarization recording should be incorporated more broadly into clinical and preclinical research, to integrate this important pathophysiological concept into our framework of energy metabolism in acute brain injury.

### 8.4 | Why should we use metabolic network modelling to study metabolic regulation of epigenetics in epilepsy?

Flux balance analysis is a computational approach used to analyze metabolic network models that contain all known metabolic reactions in a determined cell-type, multicellular system, tissue, or organism. These genome-scale metabolic network models also contain all the genes associated with each enzymatic or transport reaction (O’Brien et al., 2015; Orth et al., 2010). Flux balance analysis simulates the flow of metabolites through the network and allows identifying the metabolic reactions or pathways that can be regulated to maximize a previously defined metabolic objective (phenotype of interest). For example, this can be a biological objective like growth/biomass production or the synthesis of a metabolite. The simulation then uses experimentally determined metabolite measurements, allowing calculations to be biologically coherent, i.e., within the stoichiometry of the reactions in the network, which is a fundamental advantage over theory-only-based models (Orth et al., 2010).

In the context of brain energy metabolism and function, genome-scale models of the metabolic network formed by the neuron and astrocyte in the brain have been constructed and updated (reviewed in (Sertbas & Ulgen, 2018)). Key steps in the construction of these models are the curation to only include reactions and genes that are specific to the cell type of interest, where these data come from experimental transcriptomic databases, plus manual curation of the literature. Models are then validated with empirical metabolite measurements (Lewis et al., 2010). Therefore, although this is a computational approach, the amount of experimental data used for its construction results in models that can robustly represent the biological processes of interest. Furthermore, these have been used to simulate metabolic interactions between the neuron and the astrocyte in neurological diseases, such as Alzheimer’s disease (Lewis et al., 2010), Parkinson’s and Huntington’s disease, and schizophrenia (Sertbaş et al., 2014), identifying genes and pathways that could contribute to their physiopathology.

It is therefore of interest to use metabolic network modeling and flux balance analysis to study neurological diseases like epilepsy, where approximately 30% of patients are refractory to available pharmacological treatment, and a better understanding of the disease is required to develop new therapeutic approaches (Rho & Boison, 2022). Seizure activity has a high energy demand to maintain neuronal hyperactivity, which eventually causes severe cellular and mitochondrial dysregulation (Rho & Boison, 2022). Interestingly, both murine epilepsy models (Berger et al., 2019; Dębski et al., 2016; Kobow et al., 2013) and patients with epilepsy (Martins-Ferreira et al., 2022) present with an overall increase in DNA methylation, but the mechanisms of the interplay between metabolic and epigenetic changes in epilepsy is only starting to be addressed. For example, the administration of a ketogenic diet, known to reduce seizure frequency, induces a global decrease in DNA methylation levels in rats, mice, and epilepsy patients (Lusardi et al., 2015; Pedersen et al. 2022). However, which specific pathways cause DNA hypermethylation in epilepsy remains unknown. Given that this change can affect global gene expression, it could play an important role in both epileptogenesis and disease progression.

Flux balance analysis could be a valuable tool to identify which metabolic pathways altered in epilepsy are candidates to contribute to DNA hypermethylation. A possible approach would be to define as metabolic objectives the maximization of glutamatergic neurotransmission and S-adenosylmethionine synthesis (for examples on how to define the neurotransmission objective, readers can refer to (Acevedo et al., 2023; Sertbaş et al., 2014)). S-Adenosylmethionine is the sole substrate for DNA and histone methylation, and reduced S-adenosylmethionine levels have been shown to decrease DNA methylation levels (Ouyang et al., 2020). Performing flux balance analysis using a neuron–astrocyte metabolic network model would allow identifying the genes and pathways that most contribute to S-adenosylmethionine synthesis during active neurotransmission. Then, transcriptomic or proteomic data of patients with epilepsy can be integrated into the analysis to perform reporter metabolite analysis and reporter pathway analysis to determine if the identified candidates are also altered in epilepsy (Sertbaş et al., 2014).

There are several advantages to using flux balance analysis to study a neurological disease like epilepsy. First, healthy brain, cell-type specific metabolite measurements can be used, and these are already available (Sertbaş et al., 2014; Shichkova et al., 2021). Second, it is possible to use high-throughput gene expression data generated from whole tissue, also using pre-existing data, as cell-type specific databases for many neurological diseases have not been generated. Finally, by changing the metabolic objectives and gene expression databases, this analysis can be performed for other neurological diseases and metabolic objectives. The identified genes and metabolic pathways are selected using an unbiased method that is partly based on experimental measurements and can significantly narrow down the number of candidates generated by transcriptomics or proteomics databases. Furthermore, it is possible to integrate flux balance analysis with other types of network analyses, like graph-based analyses, which address the structure and connectivity within the network, as well as the use of different omics databases, depending on the biological question being addressed (Acevedo et al., 2023).

However, the method does have limitations. For example, while available healthy brain metabolite measurements can be used, cell-type-specific measurements specific for the disease being studied would deliver better results, but these are not available for all diseases and would need to be generated. Results would also be more accurate if the omics data were cell-type-specific. In spite of these limitations, the proposed computational analyses are a cost-effective method to make an unbiased selection of metabolic pathways of interest for brain function in health and disease, but that needs to be followed up with experimental approaches.

### 8.5 | How do activated microglia affect neuroenergetics and brain function?

Immunometabolism is an emerging field exploring the adaptations of cellular metabolism in macrophages, including microglia (brain-resident macrophages), during inflammation. Microglia become activated during various inflammatory processes in the brain, which often includes microglial proliferation (local increase in the microglial cell population), metabolic reprogramming, and release of cytokines and oxidants such as superoxide and nitric oxide (Bernier et al., 2020; Kann et al., 2022). Activation of microglia by bacterial lipopolysaccharide through Toll-like receptor 4 and by the lymphocytic cytokine interferon-gamma, for example, associates with substantial increases in aerobic glycolysis (use of glucose and release of lactate), accumulation of certain Krebs cycle metabolites such as itaconate, and decreases in mitochondrial respiration (inhibition of complex II by itaconate and inhibition of complex IV by nitric oxide) in microglia (Castillo et al., 2021; Chausse et al., 2020; Gimeno-Bayón et al., 2014). However, the details of metabolic reprogramming and the links to adaptations in immune functions in activated microglia are incompletely defined. Moreover, the impact of microglial lactate and oxidant release on neuronal energy metabolism, synaptic transmission, neural excitation–inhibition balance and, ultimately, neuronal survival has just begun to be explored (Hollnagel et al., 2020; Kann et al., 2022; Kealy et al., 2020; Xiang et al., 2021). This task is challenging because the diverse subtypes of excitatory neurons and inhibitory interneurons differ a lot in electrophysiological and energetic properties, including the role in information processing in cortical networks (Kann, 2016). Future research may address these issues and provide mechanistic insights into the pathophysiology underlying sickness behaviour, central fatigue, and cognitive impairment associated with, for example, systemic inflammation, multiple sclerosis, and neurodegenerative diseases.

### 8.6 | Why do activated microglia require aerobic glycolysis?

When exposed to pro-inflammatory stimuli, microglia become activated and respond with an increased flux of glucose to lactate despite available oxygen, i.e., with an increase in aerobic glycolysis. Blocking this increase suppresses microglial pro-inflammatory responses, and does so at the transcriptional level (Bernier et al., 2020; Cheng et al., 2021; Li et al., 2018; Shen et al., 2017). These observations raise three related questions: Why does this increase in aerobic glycolysis occur? By what mechanism is glycolytic flux linked to pro-inflammatory transcriptional events? Can this process be manipulated to suppress microglial activation?

The first of these questions is the deepest and most difficult. One potential explanation for increased aerobic glycolysis in activated microglia is to generate additional ATP for pro-inflammatory processes; however, there is no evidence that pro-inflammatory responses require substantially more energy demand than other microglial responses. Could the aerobic glycolysis be used to increase the speed of ATP production? That strategy could be useful when energy needs rise precipitously for short periods of time, such as with rapid muscle contraction, but it is difficult to see how it would benefit during a prolonged inflammatory response. Is it possible that the aerobic glycolysis is used to produce lactate, for consumption by other cells such as neurons? There is generally no paucity of available energy substrates at sites of inflammation, and aerobic glycolysis also occurs in activated immune cells outside the central nervous system (Bustamante et al., 2018; Goth, 1959). Could accelerated glycolysis serve to compensate for dysfunction of microglial mitochondria under the oxidative stress conditions of inflammation? Potentially (van Horssen et al., 2019), but this leaves open the question as to why blocking the increased glycolysis affects pro-inflammatory changes at the transcriptional level. More likely is that we simply do not yet know why this occurs. Of note, aerobic glycolysis also occurs in many cancer cells, where it is termed the Warburg effect (Warburg et al., 1927). Though first described nearly 100 years ago, there still is no generally accepted explanation as to why most cancer cells preferentially utilize aerobic glycolysis (DeBerardinis & Chandel, 2020).

The second question, as to how glycolytic flux is linked to pro-inflammatory transcriptional events, is more tractable. Activated microglia upregulate many pro-inflammatory genes, particularly those regulated by the transcription factor NF-κB, and this increase is strikingly attenuated when glycolytic flux is restricted (Bernier et al., 2020; Cheng et al., 2021; Shen et al., 2017). Evidence supports at least three different mechanisms by which glucose flux may be linked to transcriptional events. One of these is via the NADH-sensitive transcriptional co-repressor, CtBP (C-terminal binding protein), which responds to increases in cytosolic NADH (as occurs with accelerated glycolytic flux) by enhancing NF-κB transcriptional activity (Shen et al., 2017). A second mechanism is via the NLRP3 (NACHT, LRR, and PYD domains-containing protein 3) inflammasome, which has been shown to be influenced by glycolytic flux by an as-yet-unknown process (Moon et al., 2015; Xie et al., 2016). The third mechanism involves a protein phosphorylation effect of hexokinase-2, whereby high glucose levels promote dissociation of hexokinase-2 from mitochondria and its subsequent phosphorylation of IκBα to promote activation of NF-κB (Guo, Tong, et al., 2022). The relative importance of these differing signalling pathways remains to be determined, but the involvement of signalling pathways coupling glucose metabolism to transcriptional events suggest that the links between aerobic glycolysis and microglial activation extend beyond simply ATP production.

Given the role of microglial activation in brain injury, can the requirement for aerobic glycolysis by microglia be targeted to treat neurological disease? Numerous approaches to this end are currently being explored. Of note, microglia (and other immune cells) use predominantly hexokinase-2 rather than the hexokinase-1 isoform used by most other cell types, thus presenting a way to selectively reduce the microglial glycolytic rate. Recent studies have begun to exploit this fact (Cheng et al., 2021; Hu et al., 2022; Leng et al., 2022; Li et al., 2018). Whether this approach might have advantages over other means of suppressing microglial activation remains an open question with significant therapeutic importance.

## 9 | METABOLIC CONTROL UNDER EXTREME CONDITIONS

### 9.1 | How does the CNS modulate the circannual rhythm of metabolism in mammals?

Mammalian hibernators show the greatest metabolic and thermoregulatory flexibility of all mammals. These animals display seasonal obesity, reversible loss of insulin sensitivity, and extended periods of ketosis during episodes of prolonged torpor to survive resource limitations. By expressing torpor, a number of species in a wide range of orders, from monotremes to primates, save energy by suppressing thermogenesis and downregulating metabolism through poorly defined mechanisms (Buck & Barnes, 2000; Carey et al., 2003; Dausmann et al., 2004; Tupone et al., 2017). In obligate hibernators such as the arctic ground squirrel (*Urocitellus parryii*) hibernation torpor, an extension of sleep, is expressed according to an endogenous circannual rhythm. Seasonal changes in daylight entrain the rhythm to 12 months. Without changing daylength, animals free-run on an 8-to 9-month cycle (Olson et al., 2013). Neither short days, food restriction, nor cold ambient temperature is needed to induce hibernation in obligate hibernators, although low ambient temperatures enhance the depth and duration of torpor. Body temperature in humans show similar, albeit much less, extreme decreases in body temperature and increases in sleep in winter (Harding et al., 2019; Yetish et al., 2015), suggesting that mammalian hibernators offer an exaggerated expression of circannual rhythms that may also be expressed in other mammals including humans. Whether CNS adenosine is responsible for the seasonal change that facilitates the transition of sleep into torpor is a burning question that, when answered, is expected to define mechanisms that are fundamental to all mammalian metabolism, thermoregulation, and sleep.

The circannual pattern of hibernation in ground squirrels is described by a two-switch model (Epperson et al., 2011). One switch initiates transition between summer and winter. Superimposed on the winter phenotype is a switch that initiates torpor/arousal cycles characteristic of hibernation where torpor is interrupted every 2–3 weeks by 12–24 h of interbout arousal. Transition to the winter phenotype is seen first as a pre-hibernation fall, fattening period. During the pre-hibernation period beginning 6 weeks prior to the first bout of torpor, animals show a 1–2°C decrease in euthermic body temperature and increased sleep (switch 1). This is followed by abrupt and dramatic decreases and increases in metabolic rate and body temperature that mark the torpor/arousal cycle (switch 2; Chmura et al., 2022). During the winter season, these animals are also less sensitive to wake promoting effects of adenosine receptor antagonists and more sensitive to torpor-inducing effects of adenosine receptor agonists (Jinka et al., 2011; Walker et al., 1981).

Does CNS adenosine drive the fall transition? Adenosine is an inhibitory neuromodulator and retaliatory metabolite that contributes to homeostatic sleep drive and inhibits thermogenesis through receptor-mediated and other mechanisms (Boison et al., 2011; Province et al., 2020). Animals enter torpor through sleep and emerge from torpor into sleep with limited wake time during interbout arousal. If we consider time spent sleeping and lowered body temperature as readouts of CNS A_1_ adenosine receptor activation, we see greater activation in winter than in summer. Walker et al. (1981) showed increased sleep time in golden mantel ground squirrels in winter and lower sensitivity to adenosine receptor antagonists. Euthermic body temperature is lower in winter with increased sensitivity to adenosine agonist-induced decreases in body temperature and torpor (Boison et al., 2011; Frare et al., 2019; Olson et al., 2013). These data support the model of a seasonal waxing and waning in purinergic signalling, that when entrained to changing daylight is at a maximum during the winter season. An intracerebroventricular injection of the A_1_ adenosine receptor agonist N^6^ cyclohexyl adenosine into the lateral ventricle of arctic ground squirrels in summer induces a small decrease in metabolic rate and body temperature. The same dose administered to the same animals produces a response indistinguishable from spontaneous torpor in a third of the animals in fall and in all of the animals in mid-winter (Jinka et al., 2011). The winter response is associated with increased inhibitory influence of N^6^ cyclohexyl adenosine in thermoregulatory regions (the median preoptic nucleus and raphe pallidus), but not in sleep-active regions (Frare et al., 2019). So what produces this change in purinergic signaling?

At the receptor level, we found a small increase in efficacy of N^6^ cyclohexyl adenosine in the hypothalamus and hippocampus in winter using GTPγS binding, but the magnitude of change was small relative to the magnitude of change in response to N^6^ cyclohexyl adenosine in vivo (Frare et al., 2019). By contrast, large changes in plasma adenosine concentrations were found in the fall and during each bout of interbout arousal throughout the hibernation season. Plasma adenosine levels gradually increase during the pre-hibernation season, with about a 3-fold increase in plasma adenosine 1 month prior to the first torpor bout and nearly a 5-fold increase in plasma adenosine concentrations 1 week prior to the first torpor bout (unpublished data). A winter decrease in adenosine kinase expression was found in tanycytes that line the third ventricle. Although still speculative, these findings contribute to a model whereby adenosine kinase in tanycytes regulates seasonal changes in extracellular concentration of adenosine underlying the seasonal expression of hibernation. The seasonal increase in efficacy of intracerebroventricular N^6^ cyclohexyl adenosine emphasizes a central site of action. However, an increase in plasma concentrations of adenosine draws attention to peripheral sites that may contribute to energy conservation, such as bradycardia and inhibition of lipolysis (Borea et al., 2018). Elevated plasma concentrations of adenosine also beg the question as to whether CNS adenosine originates from a peripheral source.

In summary, a burning question in brain energy metabolism is what drives the seasonal expression of hibernation? The adenosine model of hibernation suggests that a seasonal increase in the gain of purinergic signaling may be responsible, but, if so, how is the gain increased and regulated? An increase in plasma levels of adenosine suggests that whole body metabolism may contribute to metabolic down-regulation. Tanycytes may play a permissive role to enable diffusion of adenosine from blood and CSF into the brain parenchyma. Although humans do not hibernate, we do sleep longer and we have slightly lower body temperature during the winter. Hibernation may be an exaggerated example of how a ubiquitous metabolite and neuromodulator like adenosine regulates energy homeostasis through an influence on thermoregulation with subsequent effects on sleep and peripheral metabolism.

## 10 | CONCLUSION

This set of vignettes from researchers in the field of brain energy metabolism poses just a few of the questions that still remain to be answered about how the brain fuels its function, and how its activity may change the resulting metabolic response and possibly even overall brain health. Major future directions include the following: understanding the metabolic interactions between difference cell types in the brain, including (largely unstudied) oligodendrocytes, as well as metabolic compartmentation within cells; how these interactions change with activation or disease states; the emerging roles of post-translational modifications to enzymes; understanding the roles of glycolysis vs oxidative phosphorylation; the role(s) of glycogen; changes in metabolism due to sex, just to name a few.

The evolution of novel and powerful tools to study metabolism will aid this journey, enabling greater and more in-depth exploration of brain energy metabolism.

## Figures and Tables

**TABLE 1:** List of published studies from which metabolic fluxes were derived for use in Figure 2 (fluxes with associated standard deviation in μmol/g/min). These studies were selected for having independently determined *V*_PC_, *V*_NT_, and TCA cycle rates in neurons (*V*_TCA_^n^) and astrocytes (*V*_TCA_^a^).

	V_NT_	V_PC_	CMR_glc(ox)_
Human			
Gruetter et al. (2001)	0.17 ± 0.05	0.09 ± 0.02	0.36 ± 0.08
Rat			
Choi et al. (2002) (isoelectricity)	0.04 ± 0.01	0.03 ± 0.01	0.32 ± 0.07
Öz et al. (2004) (awake)	0.57 ± 0.21	0.14 ± 0.03	0.84 ± 0.17
Duarte et al. (2011)	0.11 ± 0.01	0.069 ± 0.004	0.39 ± 0.02
Jeffrey et al. (2013)	0.12 ± 0.05	0.07 ± 0.02	0.38 ± 0.08
Duarte and Gruetter (2013) ^a^	0.24 ± 0.01	0.092 ± 0.005	0.47 ± 0.02
Lanz et al. (2014)	0.15 ± 0.01	0.09 ± 0.01	0.41 ± 0.06
Sonnay et al. (2016) (rest)	0.070 ± 0.014	0.064 ± 0.005	0.45 ± 0.02
Sonnay et al. (2016) (stimulation)^b^	0.137 ± 0.027	0.069 ± 0.011	0.52 ± 0.05
Sonnay et al. (2017) (isoelectricity)	0.092 ± 0.023	0.023 ± 0.004	0.32 ± 0.01
Girault et al. (2019)	0.20 ± 0.02	0.09 ± 0.01	0.37 ± 0.03
Tree shrew			
Sonnay et al. (2017) (rest)	0.19 ± 0.023	0.074 ± 0.008	0.43 ± 0.02
Sonnay et al. (2017) stimulation)^b^	0.23 ± 0.040	0.066 ± 0.006	0.49 ± 0.02
Mouse			
Lai et al. (2018)	0.084 ± 0.008	0.041 ± 0.003	0.38 ± 0.02

a Data included labelling of GABA and was modelled with a 3-compartment model (other studies included only glutamate, glutamine, and/or aspartate).

b Used only in panel D.

## Data Availability

Data sharing is not applicable to this article as no new data were created or analyzed in this study.

## Abbreviations:

ACOT: acyl-CoA thioesterase
ACSS1: gene encoding acetyl-CoA synthetase 2
ACSS2: gene encoding acetyl-CoA synthetase 1
AD: Alzheimer’s disease
DDHD2: phospholipase DDHD domain-containing protein 2
FA: fatty acid
FDG-PET: fluorodeoxyglucose positron emission tomography
GLAST: glutamate aspartate transporter
GLT-1: glutamate transporter-1
MCT1: monocarboxylate transporter 1
MCT2: monocarboxylate transporter 2
NMN: nicotinamide mononucleotide
NMNAT: nicotinamide mononucleotide adenylyl transferase
SIRT: silent information regulator
SLC13A5: sodium-dependent citrate transporter.
